# Quorum sensing regulators AphA and HapR differentially regulate fluvibactin biosynthesis in *Vibrio fluvialis*

**DOI:** 10.3389/fmicb.2026.1829491

**Published:** 2026-05-04

**Authors:** Qian Cheng, Yu Han, Kunkun Wang, Saisen Ji, Xiaorui Li, Aiping Qin, Yue Xiao, Weili Liang, Biao Kan

**Affiliations:** 1 National Key Laboratory of Intelligent Tracking and Forecasting for Infectious Diseases, National Institute for Communicable Disease Control and Prevention, Chinese Center for Disease Control and Prevention, Beijing, China; 2 Clinical Research Center, Chongqing Public Health Medical Center, Chongqing, China

**Keywords:** AphA, HapR, iron-limited conditions, quorum sensing, *Vibrio fluvialis*

## Abstract

**Introduction:**

Siderophores are secondary metabolites with high affinity and specificity for iron and play a crucial role in iron acquisition. *Vibrio fluvialis*, an emerging foodborne opportunistic pathogen of increasing concern, produces the catechol siderophore fluvibactin. Quorum sensing (QS) is a process of bacterial communication vital for many biological processes. To date, the genes responsible for fluvibactin biosynthesis and the role of quorum sensing (QS) in its regulation remain unclear.

**Methods:**

In this study, we characterized the gene cluster involved in fluvibactin biosynthesis and investigated how the QS regulators AphA at low cell density (LCD) and HapR at high cell density (HCD) regulate this process. Gene expression, promoter activity, and protein–DNA interactions were analyzed using qPCR, reporter assays, electrophoretic mobility shift assays (EMSA), DNase I footprinting, and 5′ rapid amplification of cDNA ends (RACE). Also, animal experiments were conducted with a mutant which fails to biosynthesize fluvibactin.

**Results:**

We identified a fluvibactin biosynthesis gene cluster consisting of 16 genes organized into 9 transcriptional units, which are activated under iron-deficient conditions. Fluvibactin production was increased in the Δ*aphA* mutant at LCD and decreased in the Δ*hapR* mutant at HCD compared to the wild type. Consistently, the expression and promoter activities of representative genes (*vfbH*, *vfbA*, *vfbCE* and *vfbB*) were repressed by AphA at LCD and activated by HapR at HCD. Direct binding of AphA and HapR to the promoters of these genes was confirmed. Additionally, we demonstrated that fluvibactin production was associated with increased bacterial loads in infected mice.

**Discussion:**

These findings provide insights into the genetic basis of fluvibactin biosynthesis and its regulation by QS in *V. fluvialis*. This study enhances our understanding of the pathogenicity and environmental adaptability of this organism under iron-limited conditions and contributes to the knowledge of catechol siderophores in *Vibrios*.

## 1 Introduction

Iron, an essential trace element for virtually all bacteria, is an important cofactor required for enzymes to catalyze redox reactions involved in key cellular, metabolic and biosynthetic processes (Li et al., 2016; Kramer et al., 2020). Despite its abundance in Earth’s crust, the bioavailability of iron is generally low because the main existing form of iron, namely the oxidized ferric (Fe^3+^), is largely insoluble under common aerobic and neutral pH conditions (Li et al., 2016). To acquire sufficient iron, bacteria have evolved a variety of sophisticated strategies. One of these is the secretion of siderophores, which are secondary metabolites that scavenge iron from environmental stocks by forming soluble Fe^3+^ complexes that are then actively taken up by cells via specific receptors (Kramer et al., 2020). After being first discovered in the 1950s with the identification of mycobactin as a growth factor for *Mycobacterium johnei*, out of 500 distinct siderophores have been identified (Holden and Bachman, 2015). However, only a few of them have had their biosynthetic pathways thoroughly investigated and elucidated. The biosynthesis of siderophores involves multiple steps that coordinated by specific enzymes (Schalk, 2025). Typically, amino acids, aromatic rings and small carboxylic acids are built into a peptidic precursor molecule, which is subsequently assembled into the final siderophore structure by non-ribosomal peptide synthetase (NRPS) or polyketide synthase (PKS). The mature siderophore molecule is then secreted into extracellular environment, followed by chelating iron and importing the siderophore-Fe^3+^ complexes back into cells via specific transport systems (Schalk, 2025). Based on the chemical groups involved in iron binding, bacterial siderophores can be classified into different types, including catecholate, hydroxamate, phenolate, carboxylate and mixed-type (Kramer et al., 2020). Often, bacteria can produce two or three different siderophores, which possess the ability to exploit siderophores that have been produced by other bacteria (xenosiderophores) (Schalk, 2025).

*Vibrios*, like other bacterial species, have evolved to produce different types of siderophores to acquire iron when iron is limiting (Payne et al., 2016). Catechol siderophores, which contain 2,3-dihydroxybenzoic acid (DHBA) as the iron-chelating moiety, are common in *Vibrio* spp. The catechols, which are generally in linear structures, show different backbones and have different numbers and types of amino acids linked (Payne et al., 2016). For example, *Vibrio cholerae* produces vibriobactin, which has three catechol groups attached a norspermidine (NSPD) backbone (Griffiths et al., 1984). The vibriobactin biosynthesis mechanisms and pathway have been determined. The intermediate in vibriobactin biosynthesis, DHBA, is biosynthesized from chorismate by three enzymes, VibA, VibB and VibC, which have the same functions as their *Escherichia coli* homologs EntA, EntB and EntC (Wyckoff et al., 1997). DHBA then together with L-threonine and NSPD form the precursor, from which VibF, a NPRS, together with vibriobactin synthase proteins VibE, VibH and VibB are assembled into vibriobactin (Butterton et al., 2000; Keating et al., 2000a, 2000b; Wyckoff et al., 2001). The genes encoding vibriobactin synthesis are located in two separate clusters on the large chromosome. Specifically, the genes for the synthesis of DHBA, *vibABC*, the genes for subsequent steps in biosynthesis, *vibD*, *vibE* and *vibH*, and the genes for catechol transport, *viuPDGC* are found in one cluster (Wyckoff et al., 1997; Keating et al., 2000b; Wyckoff et al., 2001). The gene for NRPS VibF, *vibF*, the genes for vibriobactin transport, *viuA*, and utilization, *viuB*, are found in another cluster (Butterton et al., 2000; Keating et al., 2000a). Like *V. cholerae*, *V. fluvialis* and *V. vulnificus* also produce catechol siderophores, fluvibactin and vulnibactin, which are structurally related to vibriobactin (Simpson and Oliver, 1983; Yamamoto et al., 1993; Okujo et al., 1994). The *V. anguillarum* O1 serotype produces a mixed catechol and hydroxamate siderophore, anguibactin (Actis et al., 1986), which is also produced by *V. harveyi* (Naka et al., 2013). *V. anguillarum* O1 strains that lack the anguibactin plasmid and strains of some other *V. anguillarum* serotypes synthesize vanchrobactin, which is a catechol siderophore distinct from anguibactin (Lemos et al., 2010). Unlike the above *Vibrio* spp., *V. parahaemolyticus* produces a carboxylate siderophore (Yamamoto et al., 1994) while *V. mimicus* and *V. hollisae* (reclassified as *Grimontia hollisae*) (Thompson et al., 2003) produce a hydroxamate siderophore (Moon et al., 2004; Suzuki et al., 2006). Some marine *Vibrio* spp. can produce amphiphilic siderophores (Martinez et al., 2003; Vraspir et al., 2011).

Quorum sensing (QS) is a process of bacterial communication that coordinates various physiological activities by producing, releasing, detecting and responding to chemical signal molecules called autoinducers (AIs) (Miller and Bassler, 2001; Waters and Bassler, 2005). QS is involved in the regulation of siderophore production and the accumulation of AIs can inhibit or promote the biosynthesis of siderophores (Stintzi et al., 1998; Lilley and Bassler, 2002; Wen et al., 2012). QS was first found to regulate siderophore biosynthesis in *Pseudomonas aeruginosa*, with the mutations of *lasR*, the gene encoding the QS regulator LasR, and of *lasI*, the gene encoding LasI for PAI-1 production, repressing the biosynthesis of the catecholate-hydroxamate siderophore pyoverdine (Stintzi et al., 1998). In *Burkholderia cepacia*, the mutations in *cepR* and *cepI*, homologs of *luxI*/*luxR* in *V. fischeri*, hyperproduced siderophore ornibactin (Lewenza et al., 1999). In *V. vulnificus*, iron and QS are shown to coordinately regulate the expression of *vvsAB*, which encode a NRPS required for vulnibactin synthesis (Wen et al., 2012). In *V. harveyi*, the phosphorylated response regulator LuxO together with σ^54^ can activate the biosynthesis of siderophores (Lilley and Bassler, 2002). In *Vibrio* species including *V. harveyi*, *V. cholerae* and *V. fluvialis*, AphA and LuxR/HapR are two master QS regulators, with AphA functioning at low cell density (LCD) and LuxR/HapR functioning at high cell density (HCD) (Lenz et al., 2004; Wang et al., 2013; Ball et al., 2017). These two QS regulator are produced in reciprocal gradients and exert repressive effect toward each other (Rutherford and Bassler, 2012).

*V. fluvialis* is a novel foodborne pathogen that can cause cholera-like acute diarrhea and various extraintestinal infections (Igbinosa and Okoh, 2010; Ramamurthy et al., 2014). *V. fluvialis* produces catechol siderophore fluvibactin, which is structurally similar to vibriobactin in containing a linear, NSPD backbone with the iron-binding moieties linked directly or through threonine (Yamamoto et al., 1993). However, different from the structure of vibriobactin, only one of the three DHBA moieties in fluvibactin is linked to NSPD by forming an oxazoline ring with threonine, while the other two are directly linked to NSPD (Yamamoto et al., 1993). Fluvibactin has been shown to be responsible for the antibacterial activity of *V. furnissii* and *V. fluvialis* against *V. anguillarum* (Giubergia et al., 2016). Also, under iron-limited conditions, the co-culture of *V. cholerae* with *V. fluvialis* can significantly promote the *V. cholerae* growth *in vitro*, suggesting that the fluvibactin piracy enhances *V. cholerae* survival (Byun et al., 2020). To date, the gene cluster responsible for fluvibactin biosynthesis have not been characterized and the regulatory mechanisms of fluvibactin biosynthesis, for example, by QS, are unknown.

In this study, we characterized the gene cluster for fluvibactin biosynthesis and the regulatory mechanisms of AphA and HapR, the master QS regulators at LCD and HCD, respectively, toward fluvibactin biosynthesis. The biosynthetic gene cluster (*vfbABCDEFH*) of fluvibactin is homologous to that of vibriobactin, despite some differences in gene organization. Under iron-limited conditions, the expressions of these genes as well as the production of fluvibactin are induced. Also, the synthesis of fluvibactin increases as the cell density increases. At LCD, AphA represses while at HCD, HapR activates the promoter activities and mRNA expressions of *vfbH*, *vfbA*/*vfbCE* and *vfbB* genes, and the synthesis of fluvibactin. The AphA and/or HapR binding and the transcription sites (TSSs) are confirmed on the promoter regions of *vfbH*, *vfbA*/*vfbCE* and *vfbB*. Further, *in vivo* study showed that the synthesis of fluvibactin significantly increases the bacterial loads in infected mice. Together, our results provide insights into the gene cluster for fluvibactin biosynthesis and the regulatory mechanisms of QS regulators AphA and HapR on fluvibactin biosynthesis, which will enrich our understandings of the environmental adaptability and pathogenicity of *V. fluvialis* in natural reservoirs with low iron availability, as well as the characteristics and regulatory patterns of siderophores of *Vibrios*.

## 2 Materials and methods

### 2.1 Bacterial strains, culture conditions and plasmids

All bacterial strains and plasmids used in this study are listed in Supplementary Table S1. *V. fluvialis* strain 85003, a clinical isolate from a patient with diarrhea (Wang et al., 2013; Zheng et al., 2022) (Accession: PRJNA744458; Assembly: GCA_019854215.1, Strain: VF057) and has been used in our previous studies (Huang et al., 2017; Liu et al., 2021; Huang et al., 2022; Zhang et al., 2024), was served as the wild-type (WT) strain and the *vfbACEB* in-frame deletion strain Δ*vfbACEB* was generated based on WT. *Escherichia coli* (*E. coli*) strains Top10, DH5α*λpir*, SM10*λpir* and BL21 were used for cloning (Top10 and DH5α*λpir*), conjugation (SM10*λpir*), expression and purification of the AphA and HapR proteins (BL21), respectively. All strains were grown in Luria–Bertani (LB) broth (Oxoid, Basingstoke, United Kingdom) containing 1% NaCl at 37 °C. For growth experiments under low-iron conditions, a master mix of 2.7 M 2,2′-bipyridine (C_10_H_8_N_2_, M030625, MREDA, China) was prepared and sterilized by filtration, before adding to the growth medium at indicated concentrations. Antibiotics were used at the following final concentrations (wt/vol) as necessary: ampicillin (Amp), 100 μg/mL; chloramphenicol (Cm), 10 μg/mL for *E. coli* and 3 μg/mL for *V. fluvialis*; streptomycin (Sm), 100 μg/mL; kanamycin (Km), 50 μg/mL. Isopropyl-b-D-thiogalac-topyranoside (IPTG) was added as necessary at a final concentration of 0.4 or 0.6 mM.

### 2.2 Bioinformatic analysis of the siderophore biosynthesis genes in *Vibrio* species

The protein sequences of the gene cluster for vibriobactin biosynthesis in *V. cholerae* O395 (CP001235.1) were retrieved from NCBI database and used as the reference sequences for tblasn (a protein alignment-based nucleotide search method, https://blast.ncbi.nlm.nih.gov/Blast.cgi), in search of the homologous gene cluster for siderophore biosynthesis in 11 pathogenic *Vibrio* species (*V. vulnificus*, *V. splendidus*, *V. furnissii*, *V. fluvialis*, *V. anguillarum*, *V. harveyi*, *V. metschnikovii*, *V. campbellii*, *V. alginolyticus*, *V. mimicus* and *V. parahaemolyticus*), with the identity ≥30% and the coverage ≥50%. Homologous gene clusters were identified in eight pathogenic *Vibrio* species, with the names of representative strains and the corresponding accession numbers as follow: *V. harveyi* SB1 (CP125875.1), *V. vulnificus* NBRC 15645 (CP012882.1), *V. fluvialis* ATCC 33809 (CP014035.2), *V. furnissii* FDAARGOS_777 (CP040990.1), *V. anguillarum* PF4-E1-1 (CP031479.1) and *V. splendidus* 2_C04b (CP089203.1), *V. metschnikovii* NCTC 8443 (UHIH01000002.1), *V. campbellii* BoB-53 (CP026321.1). Clinker^1^ was used for the alignment and visualization of siderophore biosynthesis gene clusters in the above *Vibrio* strains. The classical strain *V. cholerae* O395 was selected as the primary reference because the foundational studies elucidating the vibriobactin biosynthesis pathway were predominantly conducted using this biotype (Butterton et al., 2000; Keating et al., 2000b), though the cluster is highly conserved in El Tor strains (Dziejman et al., 2002; Beyhan et al., 2006).

### 2.3 Construction of the *vfbACEB* in-frame deletion mutant

The *vfbACEB* in-frame deletion mutant Δ*vfbACEB* was generated by allelic exchange based on WT (Wu et al., 2015). Briefly, the upstream and downstream flanking fragments (about 1,000 bp for each) of the target genes were amplified using corresponding primers shown in Supplementary Table S2. The purified PCR products were stitched together and then ligated into the pWM91 suicide plasmid at XhoI-SacI sites to generate pWM91Δ*vfbACEB* recombinant plasmid using the homologous recombination kit (TransGen Biotech, Beijing), followed by transferring the pWM91Δ*vfbACEB* into DH5α*λpir*. The recombinant plasmid was then introduced into WT (the recipient) from SM10*λpir* (the donor) by conjugation. The conjugants were screened on LB agar plates containing Amp and Sm, followed by transferring the antibiotics-resistant colonies to NaCl-free LB agar plates with 10% sucrose for the second selection. The sucrose-resistant colonies were further tested for sensitivity to Amp and resistance to Sm, before being confirmed by PCR and DNA sequencing.

### 2.4 Growth curve analysis

The overnight LB cultures of *V. fluvialis* strains were diluted (1:100) into fresh LB medium supplemented with different concentrations of 2,2′-bipyridine. Triplicates of 200 μL diluted cultures were transferred to a 100-well microtiter plate and incubated at 37 °C with constant shaking at 200 rpm. The values of OD_600_ were measured every 30 min using Bioscreen (Oy Growth Curve, Finland). The average values of OD_600_ were plotted against the corresponding time points to generate growth curves.

### 2.5 Chrome azurol sulphonate assay

Chrome azurol sulphonate (CAS) assay was performed as previously described (Schwyn and Neilands, 1987). Briefly, the overnight LB cultures of *V. fluvialis* strains were diluted (1:100) into fresh LB medium supplemented with different concentrations of 2,2′-bipyridine and incubated at 37 °C with shaking at 200 rpm for 3–6 h until the values of OD_600_ reached 0.2, 0.4 or 0.8. The supernatants were collected by centrifugation. Triplicates of 100 μL supernatant were transferred to a 96-well microtiter plate and then each replicate was mixed with 100 μL modified CAS assay solution (Coolaber, Beijing). The mixtures were then equilibrated for 3 h at room temperature and the absorbance values at 630 nm (OD_630_) were measured using spectrophotometer (Infinite M200 Pro, Tecan, Austria). The uncultured fresh LB medium containing corresponding concentration of 2,2′-bipyridine was used as a negative control. The OD_630_ values of the cultured supernatants were recorded as As, while the OD_630_ values of the uncultured fresh LB medium containing corresponding concentration of 2,2′-bipyridine were recorded as Ar. The fluvibactin production unit (FPU) was calculated by the equation FPU = (Ar-As)/Ar.

### 2.6 Cross-feeding assay

Cross-feeding assay was performed as previously described (Wyckoff et al., 2015). Briefly, the overnight LB culture of Δ*vfbACEB* strain (recipient strain) was diluted (1:100) into fresh LB medium containing 270 μM 2,2′-bipyridine to inhibit the growth of Δ*vfbACEB*. The culture supernatants of donor strains (WT, Δ*aphA*, Δ*hapR*, and Δ*vfbACEB*) cultured in LB containing 200 μM 2,2′-bipyridine were collected when the cultures reached OD_600_ = 0.2 and 0.8. At each sampling point, culture supernatants from all four strains were harvested (the culture supernatants of Δ*vfbACEB* were served as the negative control of fluvibactin biosynthesis). The collected culture supernatants were centrifuged at high speed and then filter-sterilized using a 0.22 μm syringe filter to ensure the complete removal of any carryover donor cells. Then, 2 mL culture supernatant of each donor strain was mixed with 4 mL culture of the recipient strain Δ*vfbACEB*, followed by incubating at 37 °C for 6 h with constant shaking at 200 rpm. Finally, 100 μL mixed culture was serially diluted and then spread on LB agar plates with Sm for counting the CFUs (Colony forming units) of the recipient strain Δ*vfbACEB* after cross-feeding.

### 2.7 RNA extraction and quantitative real-time PCR (qRT-PCR)

The overnight LB cultures of WT, ∆*aphA* and ∆*hapR* were diluted (1:100) into fresh LB medium supplemented with 200 μM 2,2′-bipyridine and incubated at 37 °C. Pellets were collected at LCD (OD_600_ = 0.2) (WT and ∆*aphA*) and HCD (OD_600_ = 0.8) (WT and ∆*hapR*). Total RNA extraction and the following cDNA synthesis were performed as previously described (Wu et al., 2015). Three biological replicates were set for each sample. Relative expression values (*R*) were calculated by the equation *R* = 2^−(CT target − CT reference)^, where the CT is the fractional threshold cycle and the *recA* gene was used as internal reference for all reactions. RNA without reverse transcription was served as negative control and relevant primers were listed in Supplementary Table S2.

### 2.8 Luminescence activity assay

The promoter regions (approximately 300–500 bp upstream of the translation start site, encompassing the entire intergenic region and putative promoter elements) of *vfbH*, *vfbA*, *vfbCE* and *vfbB* were amplified and cloned into the pBBR*lux* plasmid, which has a promoterless *luxCDABE* operon. Relevant primers were listed in Supplementary Table S2. The fusion plasmids p*vfbH-lux*, p*vfbA-lux*, p*vfbCE-lux* and p*vfbB-lux* were individually transformed into SM10*λpir* and then separately mobilized into WT, ∆*aphA* or ∆*hapR*, respectively, by conjugation. The overnight LB cultures of WT and ∆*hapR* were diluted (1:100) into fresh LB medium supplemented with 200 μM 2,2′-bipyridine, while the overnight LB culture of ∆*aphA* was diluted (1:1,000) into fresh LB medium supplemented with 200 μM 2,2′-bipyridine (Sun et al., 2012). Triplicates of 200 μL diluted cultures of WT, ∆*aphA* and ∆*hapR* were transferred to opaque 96-well microtiter plates and incubated at 37 °C with constant shaking at 200 rpm. The values of luminescence (Lux, Integration time at 1000 ms) and OD_600_ (Absorbance at 600 nm) were measured every hour by spectrophotometer (Infinite M200 Pro, Tecan, Austria). The relative luminescence intensity RLU (Lux/OD_600_) was calculated to reflect the promoter activity as previously described (Pan et al., 2018).

### 2.9 Determination of the 5′-end of *vfbH*, *vfbA*, *vfbCE* and *vfbB* mRNAs

5′ RACE (rapid amplification of cDNA ends) was performed as previously described (Wu et al., 2015) to determine the TSSs of *vfbH*, *vfbA*, *vfbCE* and *vfbB* mRNAs. The WT strain 85003 was grown in LB containing 1% NaCl at 37 °C with agitation to OD_600_ 0.5. Total RNA was extracted as above described. Then the cDNA was generated using the SMARTer^®^ RACE 5′/3′ Kit (Takara Bio, United States) according to the manufacturer’s instructions. Briefly, the cDNA was amplified by the primary PCR using UPM (universal primer) provided in the kit and the corresponding gene specific primer (GSP), and the secondary, or “nested” PCR using UPM Short provided in the kit and the corresponding nested GSP. The PCR products obtained were gel-purified and cloned into the pRACE vector. Clones were sequenced using the primers M13-R and M13-F to determine the TSSs. Relevant primers were listed in Supplementary Table S2. The putative-35 and -10 promoter elements upstream of the experimentally confirmed TSSs were predicted according to BPROM (Prediction of bacterial promoters, http://www.softberry.com/berry.phtml?topic=bprom&group=programs&subgroup=gfindb) (Solovyev and Salamovand, 2011).

### 2.10 Expression and purification of AphA-His6 and HapR-His6

The *E. coli* strain BL21 (DE3) containing the pET*aphA* or pET*hapR* plasmid was cultured in LB medium with Km at 37 °C with constant shaking at 200 rpm. When the OD_600_ values reached 0.4–0.6, 0.4 mM and 1.0 mM IPTG were added to induce the expression of AphA and HapR, respectively. Then the culture of BL21 (DE3) containing pET*aphA* was incubated at 16 °C with constant shaking at 100 rpm for 20 h, while the culture of BL21 (DE3) containing pET*hapR* was incubated at 30 °C with constant shaking at 200 rpm for 3 h. The AphA-His_6_ and HapR-His_6_ proteins were purified by affinity chromatography Ni^2+^ resin (Thermo Fisher Scientific, United States) and concentrated by Ultra-10 centrifugal filter (Merck, Germany) according to the manufacturer’s instructions.

### 2.11 Electrophoretic mobility shift assay

The promoter regions (approximately 300–500 bp upstream of the translation start site, encompassing the entire intergenic regions) of *vfbH*, *vfbA*/*vfbCE* and *vfbB* were amplified with corresponding biotin-labeled primer pairs, with the plasmids of p*vfbH*-*lux*, p*vfbCE*-*lux* and p*vfbB*-*lux* as templates, respectively. Relevant primers were listed in Supplementary Table S2. The reaction mixture (20 μL) of 20 ng biotin-labeled probes with increasing amounts of purified AphA-His_6_ in binding buffer (1 mM MgCl_2_, 0.5 mM EDTA, 0.5 mM DTT, 50 mM NaCl, 10 mM Tris–HCl, pH = 7.5) together with 100 ng BSA and 100 ng dI-dC was incubated at 30 °C for 20 min, and then separated on a 6% native polyacrylamide gel, transferred to nylon membranes and visualized by Chemiluminescent Nucleic Acid Detection Module (Thermo Fisher Scientific, United States) according to the manufacturer’s instructions. The reaction for HapR-His_6_ was similar to that for AphA-His_6_, except that the biotin-labeled probes were 15 ng and the binding buffer (pH = 7.9) consisted of 10 mM Hepes, 100 mM KCl, 0.2 mM EDTA, 2 mM DTT and 10% [vol/vol] glycerol.

### 2.12 DNase I footprinting assay

DNase I footprinting assay was performed as previously described (Zianni et al., 2006). Briefly, the *vfbH*, *vfbA*/*vfbCE* and *vfbB* probes were generated by amplifying the promoter regions of *vfbH*, *vfbA/vfbCE* and *vfbB* using corresponding primer pairs listed in Supplementary Table S2, with high fidelity PCR kit (Sangon Biotech, Shanghai). Each probe (350 ng for *vfbH*, 590 ng for *vfbA* and 250 ng for *vfbB*) was mixed with purified AphA proteins (0 μM, 7.5 μM or 15 μM) in a 40 μL reaction mixture, followed by incubating at 37 °C for 0.5 h. After incubation, the reaction mixture was digested by 0.015 U RNase-Free DNase I (Promega, United States) and then analyzed by Applied Biosystems 3500XL DNA Analyzer (Thermo Fisher Scientific, United States) and Peak Scanner v1.0 (Thermo Fisher Scientific, United States).

### 2.13 Measurement of the visceral bacterial loads in mice

The mouse experiments were conducted in a specific pathogen-free (SPF) facility (maintained at 22 ± 2 °C with 50 ± 10% humidity) to ensure a sterile environment. Twelve six-week-old female C57BL/6 mice (Beijing Vital River Laboratory Animal Technology Co., Ltd., Beijing, China) were acclimatized to the SPF facility, with free access to sterile food and water. Six mice were set for each group. The WT and Δ*vfbACEB* strains, which had been frozen in a −80 °C freezer, were resuscitated using LB plates with Sm (100 μg/mL). Mice were administered 50 μL of Sm (400 mg/mL) via gavage to eliminate intestinal microbiota and standardize original immunological status. Sterile food and water were restored immediately after gavage. Meanwhile, single clones of WT and Δ*vfbACEB* strains were picked from LB plates into liquid LB medium with Sm (100 μg/mL) to culture overnight. The overnight LB cultures of *V*. *fluvialis* strains were then diluted (1:100) into fresh LB medium with Sm (100 μg/mL) and incubated for 5 h at 37 °C until the OD_600_ values reached 1.5. The bacterial suspensions were centrifuged at 5000 rpm for 3 min, followed by re-suspending the pellets in PBS. Mice were then infected via intraperitoneal injection with 100 μL of bacterial suspension (approximately 2 × 10^9^ CFUs). The remaining bacterial suspensions were serially diluted and then spread on LB agar plates supplemented with Sm (100 μg/mL) for counting the CFUs. At 12 h post-infection, mice were euthanised via cervical dislocation. The liver, spleen and lung tissues were harvested, weighed and homogenized with PBS. Gradient dilutions of these samples were then performed on LB agar plates containing Sm (100 μg/mL) to quantify the number of viable intracellular bacteria. The CFUs from tissue homogenates were counted, and data analysis was performed. The bacterial colony counts were measured as CFU/g. All procedures were conducted under strict aseptic conditions in accordance with institutional animal care and use guidelines.

### 2.14 Statistical analysis

Statistical analysis was performed by GraphPad Prism v8.0 with the unpaired two-tailed Student’s *t*-test. *p*-values of ≤0.05 were considered to be significant.

## 3 Results

### 3.1 Characterization of the homologous sequences of the gene cluster for vibriobactin biosynthesis in in *Vibrio fluvialis* and other pathogenic *Vibrios*

**Figure 1:**
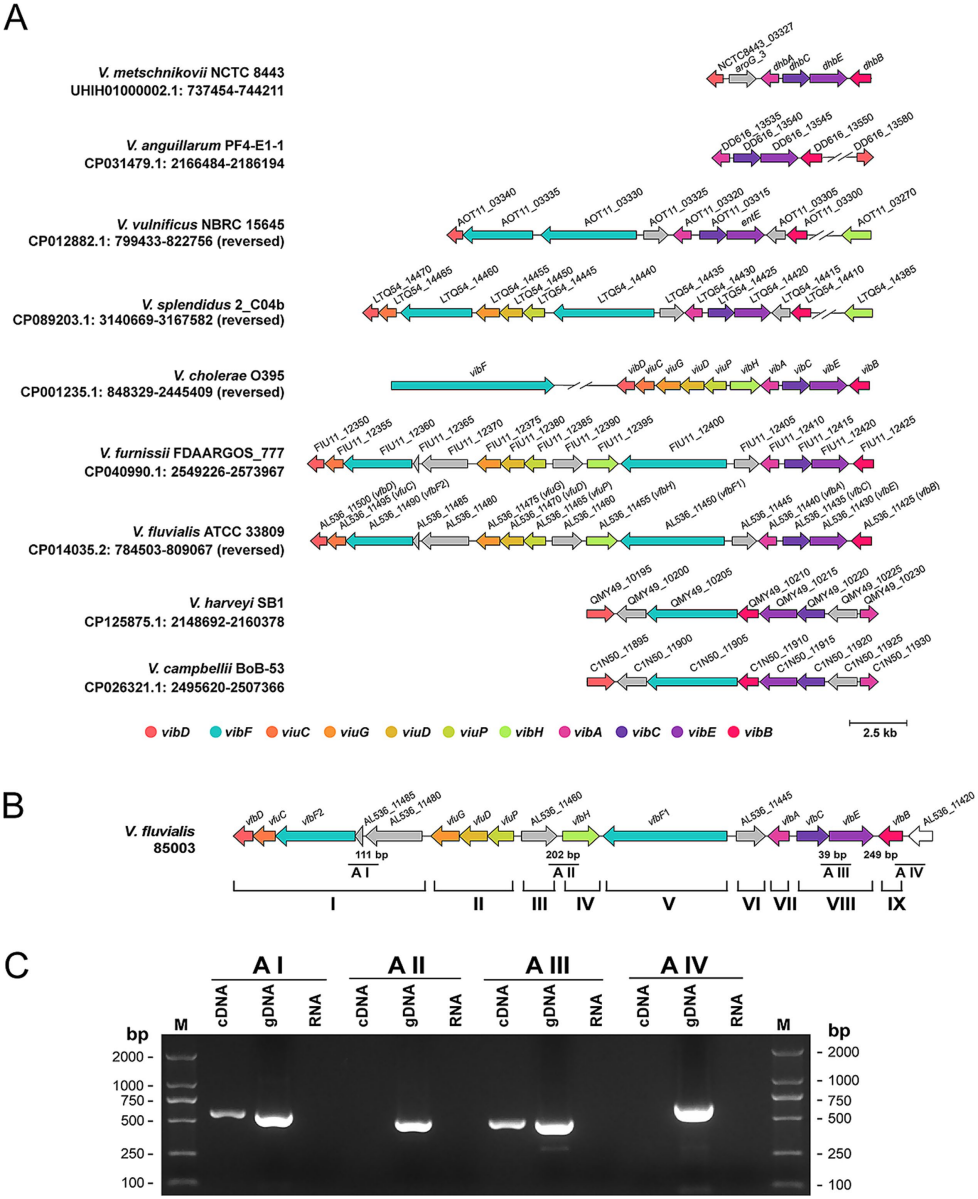
Characterization of the homologous sequences of the gene cluster for vibriobactin biosynthesis in pathogenic *Vibrios*. **(A)** Illustration of the homologous sequences of the gene cluster for vibriobactin biosynthesis and the adjacent genes for vibriobactin transport in pathogenic *Vibrios*. For the representative strain of each species (the strain names were shown next to the species names), the NCBI accession numbers and the locus_tags of homologous genes were shown. The known homologous genes for vibriobactin biosynthesis (*vibABCDEFH*) and their adjacent genes for vibriobactin transport (*viuPDGC*) in *V. cholerae* O395 were shown in different colors, while the unmatched genes in the strains of other species were shown in grey. The length scale represented 2.5 kb. **(B)** Illustration of the genes for fluvibactin biosynthesis (*vfbABCDEFH*), their adjacent genes for fluvibactin transport (*vfuPDGC*) and other genes with unknown functions (the locus_tags of genes in *V. fluvialis* 33809 were shown) in *V. fluvialis* 85003. For genes used to test for transcript units, the lengths of intergenic regions were indicated. The A I, A II, A III, and A IV indicated the primer pairs used to test for transcript units in panel **(C)**. The I–IX indicated the nine transcription units of the gene cluster for fluvibactin biosynthesis, as confirmed in panel **(C)**. **(C)** RT-PCR analysis of the transcriptional units of the gene cluster for fluvibactin biosynthesis. The cDNA indicated the complementary DNA reverse-transcribed from RNA. The gDNA (genomic DNA) and RNA (RNA without reverse transcription) were served as positive and negative controls, respectively. The M indicated marker.

Using the protein sequences of genes for vibriobactin biosynthesis (*vibABCDEFH*) and their adjacent genes for vibriobactin transport (*viuPDGC*) as references, homologous gene sequences formed in a cluster were searched in pathogenic *Vibrio* spp. (*V. vulnificus*, *V. splendidus*, *V. furnissii*, *V. fluvialis*, *V. anguillarum*, *V. harveyi*, *V. metschnikovii*, *V. campbellii*, *V. alginolyticus*, *V. mimicus*, and *V. parahaemolyticus*) through tblastn (identity ≥30%, coverage ≥50%). As the results of sequence alignment shown in Figure 1, homologous gene sequences were identified in *V. metschnikovii*, *V. anguillarum*, *V. vulnificus*, *V. splendidus*, *V. furnissii*, *V. fluvialis*, *V. harveyi* and *V. campbellii*. The composition and organization of the homologous genes in *V. fluvialis* and *V. furnissii* are highly similar. According to the names of genes for vibriobactin biosynthesis and transport, the homologous genes identified in *V. fluvialis* for fluvibactin biosynthesis and transport were named as *vfbABCDEFH* and *vfuPDGC*, respectively (Figure 1). Distinct from the single *vibF* gene for vibriobactin biosynthesis in *V. cholerae*, two homologous genes (*vfbF*1 and *vfbF*2) for fluvibactin biosynthesis in *V. fluvialis* were found. Also, the *vibF* gene is located in another cluster different from the cluster of other vibriobactin biosynthesis genes, while both the *vfbF*1 and the *vfbF*2 genes are in the same cluster as other fluvibactin biosynthesis genes. Further, compared with the gene cluster for vibriobactin biosynthesis and transport in *V. cholerae*, in *V. fluvialis*, four extra genes were identified, which are AL536_11485 and AL536_11480 between *vfbF*2 and *vfuG*, AL536_11460 between *vfuP* and *vfbH*, AL536_11445 between *vfbF*1 and *vfbA*, respectively. Therefore, although most genes showed homologies, the gene organization for fluvibactin biosynthesis in *V. fluvialis* is very different from that for vibriobactin biosynthesis in *V. cholerae*.

To further characterize the genes *vfbABCDEFH* and *vfuPDGC*, and the four extra genes (AL536_11485, AL536_11480, AL536_11460 and AL536_11445) in *V. fluvialis*, we analyzed the transcription units among them. Genes in the same orientation with intergenic regions less than 35 bp are assumed to be co-transcribed as a single transcript, while genes in the same orientation with intergenic regions more than 300 bp are assumed to be independently transcribed as different transcripts. For genes in the same orientation with intergenic regions more than 35 bp RNA but less than 300 bp, different primer pairs (A I, A II, A III and A IV shown in Figure 1B) were designed to target the corresponding regions. Then the RNA was exacted from the WT strain 85003 and reverse transcription (RT)-PCR was performed with these primers. As the results shown in Figure 1C, the genes *vfbF2*, AL536_11485 and AL536_11480 are co-transcribed as a single transcript, the genes *vfbC* and *vfbE* are co-transcribed as a single transcript, while the genes AL536_11460, *vfbH* and *vfbB* are independently transcribed as three different transcripts. Therefore, as shown in Figure 1B, we suggested that the gene cluster for fluvibactin biosynthesis can form nine transcript units (I, II, III, IV, V, VI, VII, VIII and IX).

### 3.2 Iron limitation and QS involve in fluvibactin synthesis

**Figure 2:**
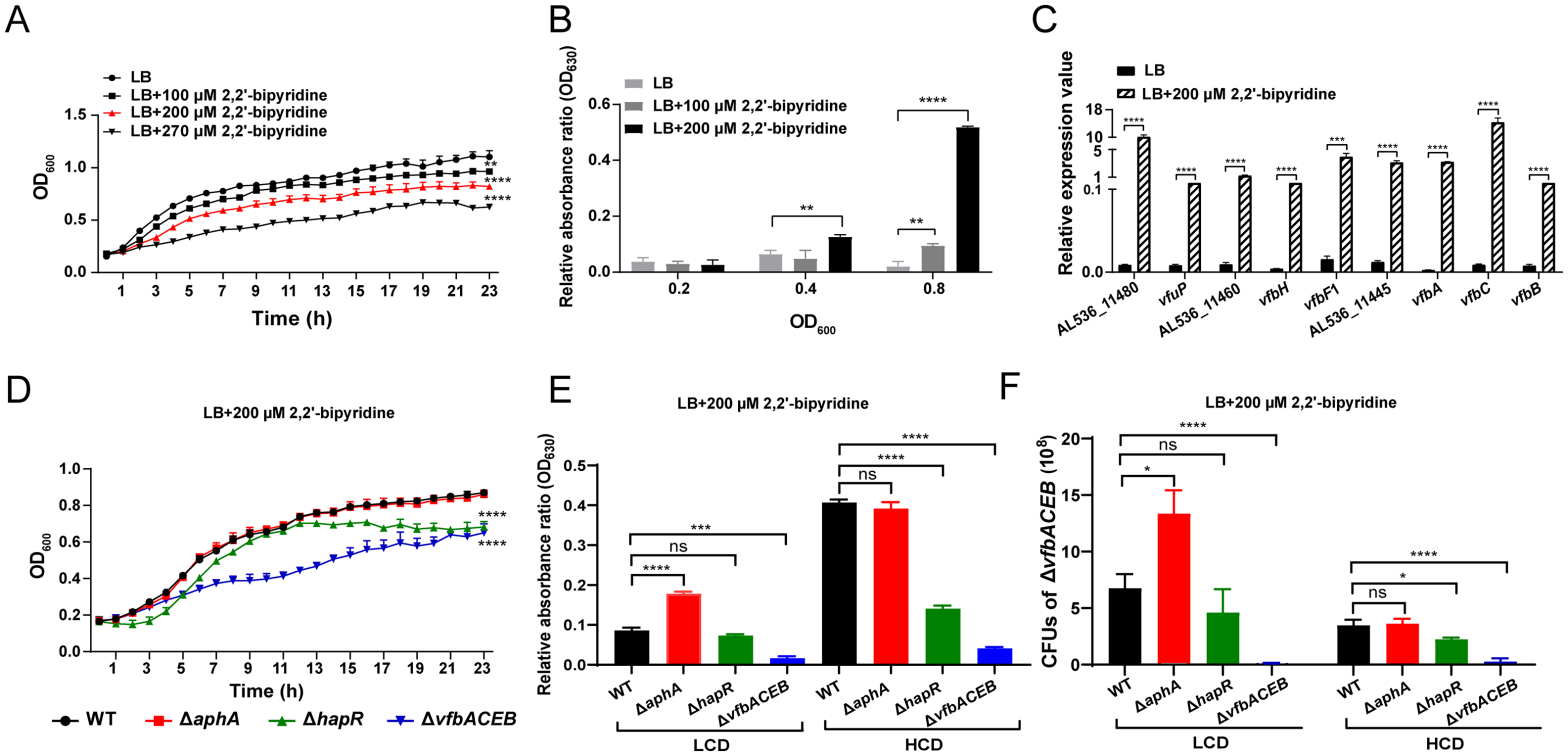
Iron limitation and QS involve in fluvibactin synthesis. **(A)** Growth of WT under different concentrations (0, 100, 200 and 270 μM) of 2,2′-bipyridine. **(B)** Fluvibactin synthesis in WT under different concentrations (0, 100 and 200 μM) of 2,2′-bipyridine and different cell density (OD_600_ = 0.2, 0.4, 0.8). Fluvibactin production was determined by CAS assay and is presented as the relative absorbance ratio (OD_630_). **(C)** Relative mRNA levels of AL536_11480, *vfuP*, AL536_11460, *vfbH*, *vfbF1*, AL536_11445, *vfbA*, *vfbC*, and *vfbB* under control LB and iron-limited (LB + 200 μM 2,2′-bipyridine) conditions at OD_600_ = 0.8. QPCR was performed to examine the relative mRNA levels and *recA* was used as internal control. **(D)** Growth of WT and mutants (Δ*aphA*, Δ*hapR*, and Δ*vfbACEB*) under LB conditions with 200 μM 2,2′-bipyridine. **(E)** Fluvibactin synthesis in WT, Δ*aphA*, Δ*hapR*, and Δ*vfbACEB* under LB conditions with 200 μM 2,2′-bipyridine. Fluvibactin synthesis was examined at both LCD (OD_600_ = 0.2) and HCD (OD_600_ = 0.8) for all strains. CAS assay was performed to examine fluvibactin synthesis. **(F)** The CFUs of Δ*vfbACEB* grown in the supernatants of WT, Δ*aphA*, Δ*hapR*, and Δ*vfbACEB* under LB conditions with 200 μM 2,2′-bipyridine. Culture supernatants from all four donor strains were collected at OD_600_ = 0.2 and 0.8. Cross-feeding assay was performed to examine the CFUs of Δ*vfbACEB* grown in different supernatants. Results represented the mean ± SD of three independent biological replicates (*n* = 3). Statistical significance was determined using an unpaired two-tailed Student’s *t*-test (^*^p < 0.05, ^**^p < 0.01, ^***^p < 0.001, and ^****^p < 0.0001).

In *Vibrios*, iron limitation prevents growth of bacteria (Payne et al., 2016; Byun et al., 2020). To determine whether the case applies to *V. fluvialis*, the growth of WT strain was examined under different concentrations (0, 100, 200 and 270 μM) of 2,2′-bipyridine, a lipid-soluble ferrous iron chelator. As shown in Figure 2A, the growth of WT was inversely proportional to the concentrations of 2,2′-bipyridine, with the growth rate decreasing by about one-third at 200 μM 2,2′-bipyridine and by about one-half at 270 μM 2,2′-bipyridine. We further examined the ability of *V. fluvialis* to produce fluvibactin under iron-limited conditions. As shown in Figure 2B, the production of fluvibactin was directly proportional to the concentrations of 2,2′-bipyridine and cell density, with the fluvibactin production being highest at 200 μM 2,2′-bipyridine and OD_600_ = 0.8 (more than 5-fold compared to LB medium without 2,2′-bipyridine addition and at OD_600_ = 0.2). We also examined the mRNA levels of representative genes (AL536_11480, *vfuP*, AL536_11460, *vfbH*, *vfbF*1, AL536_11445, *vfbA*, *vfbC* and *vfbB*) in the cluster for fluvibactin biosynthesis at 200 μM 2,2′-bipyridine, which was applied in subsequent experiments to mimic iron-limited conditions. As shown in Figure 2C, compared to control LB conditions, the mRNA levels of these genes were significantly higher (ranging from 49-fold to 1755-fold) at 200 μM 2,2′-bipyridine. Together, the results showed that iron limitation inhibits the growth of *V. fluvialis*, but enhances the synthesis of fluvibactin and the expression of genes for fluvibactin biosynthesis and their adjacent genes.

Since the synthesis of fluvibactin was influenced by cell density (Figure 2B), we investigated the effect of QS regulators AphA at LCD and HapR at HCD (Rutherford and Bassler, 2012) on the growth of *V. fluvialis* and fluvibactin synthesis under iron-limited conditions. We firstly demonstrated that *aphA* was highly expressed at OD_600_ = 0.2, which represented LCD, while *hapR* was highly expressed at OD_600_ = 0.8, which represented HCD (Figure S1). The *vfbACEB* in-frame deletion strain Δ*vfbACEB*, which cannot synthesize the intermediate DHBA for subsequent fluvibactin biosynthesis, was generated as a negative control. The growth and the fluvibactin synthesis of WT, QS regulator deletion strains Δ*aphA* (Cheng et al., 2024) and Δ*hapR* (Wang et al., 2013), as well as Δ*vfbACEB* were examined at 200 μM 2,2′-bipyridine. As shown in Figure 2D, the growth of WT and Δ*aphA* were similar, while the growth of Δ*hapR* was significantly slower and that of Δ*vfbACEB* was the slowest. As for fluvibactin synthesis, compared to WT, the amount of fluvibactin produced in Δ*aphA* increased by approximately 2-fold at LCD while that in Δ*hapR* decreased by less than 4-fold at HCD (Figure 2E). Importantly, control experiments showed that fluvibactin production in Δ*hapR* at LCD and Δ*aphA* at HCD remained comparable to WT levels, confirming the density-specific roles of these regulators. The cross-feeding assay further demonstrated the results as the supernatant of Δ*aphA* at LCD strongly stimulated the growth of Δ*vfbACEB* (the CFUs of Δ*vfbACEB* in WT supernatant/the CFUs of Δ*vfbACEB* in Δ*aphA* supernatant = 0.43), whereas the supernatant of Δ*hapR* at HCD inhibited the growth of Δ*vfbACEB* (the CFUs of Δ*vfbACEB* in WT supernatant/the CFUs of Δ*vfbACEB* in Δ*hapR* supernatant = 1.4) (Figure 2F). By contrast, the Δ*vfbACEB* treated with the supernatants of Δ*hapR* at LCD or Δ*aphA* at HCD showed similar growth as the Δ*vfbACEB* treated with the supernatants of WT at LCD or HCD, indicating the roles of AphA at LCD and of HapR at HCD. Together, these results suggested that under iron-limited conditions, QS regulator AphA represses fluvibactin synthesis at LCD, while QS regulator HapR activates fluvibactin synthesis at HCD.

### 3.3 QS regulators AphA and HapR regulate the expression of genes for fluvibactin biosynthesis

**Figure 3:**
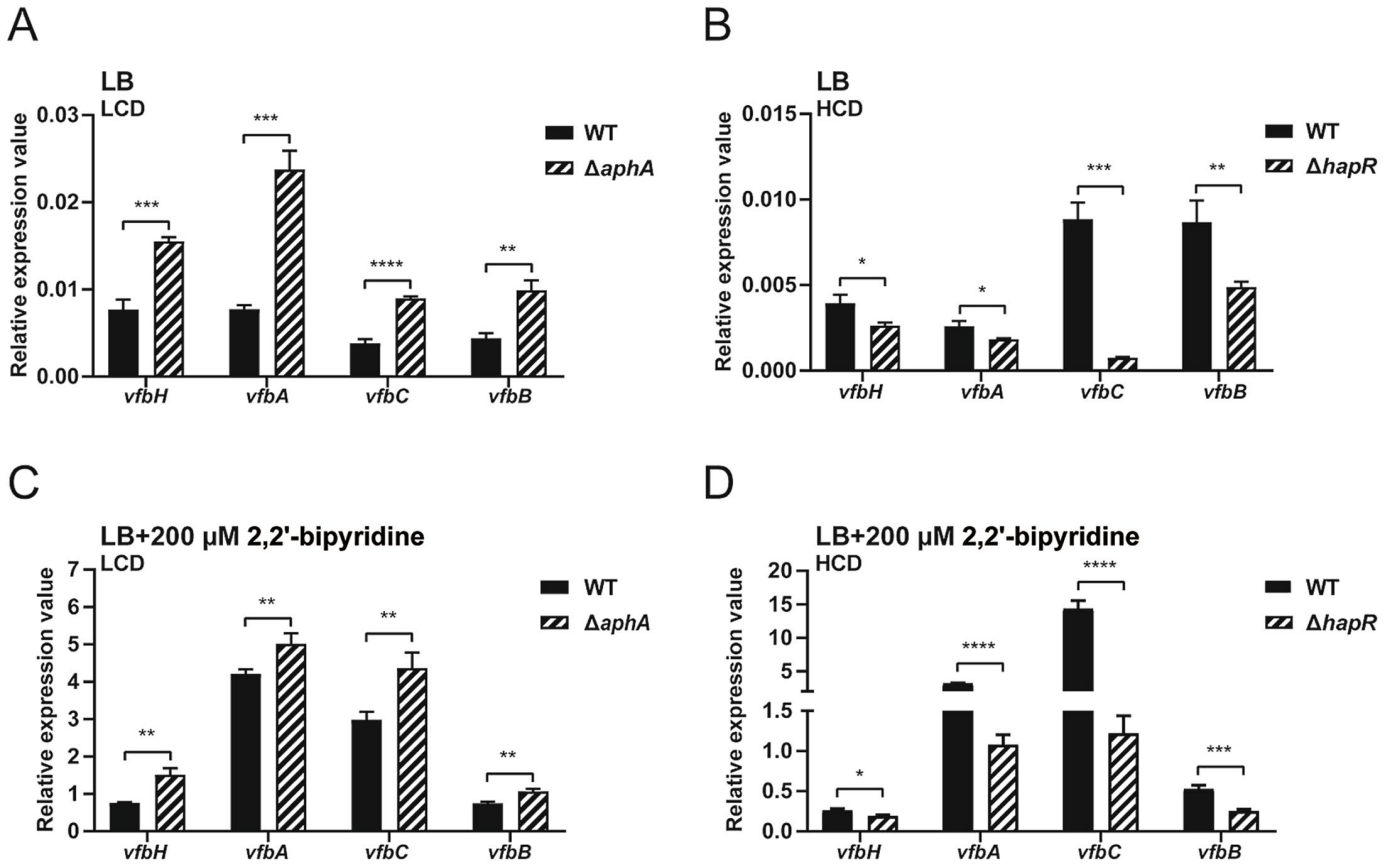
QS regulators AphA and HapR regulate the expression of *vfbH*, *vfbA*, *vfbC*, and *vfbB*. **(A,B)** Relative mRNA levels of *vfbH*, *vfbA*, *vfbC*, and *vfbB* in WT, Δ*aphA*, and Δ*hapR* under control LB conditions. **(C,D)** Relative mRNA levels of *vfbH*, *vfbA*, *vfbC*, and *vfbB* in WT, Δ*aphA*, and Δ*hapR* when 200 μM 2,2′-bipyridine was added. For Δ*aphA*, the RNA at LCD (OD_600_ = 0.2) was extracted; for Δ*hapR*, the RNA at HCD (OD_600_ = 0.8) was extracted; for WT, the RNA at both LCD and HCD was extracted. qPCR was performed to examine the relative mRNA levels and *recA* was used as internal control. Results represented the mean ± SD of three independent biological replicates (*n* = 3). Statistical significance was determined using an unpaired two-tailed Student’s *t*-test (^*^p < 0.05, ^**^p < 0.01, ^***^p < 0.001, ^****^p < 0.0001).

In *V. cholerae*, the genes *vibABC* encode for the intermediate DHAB (Wyckoff et al., 1997). Therefore, the homologous genes *vfbABC*, the adjacent gene *vfbE* in the same transcript unit as *vfbC*, and the another adjacent gene *vfbH*, were chosen and how the QS regulators AphA and HapR involve in their expression and promoter activities were investigated. As shown in Figures 3A,B, under control LB conditions, compared to WT, at LCD, the mRNA levels of *vfbH*, *vfbA*, *vfbC* and *vfbB* in Δ*aphA* were higher (ranging from 2-fold to 4-fold), while at HCD, the mRNA levels of these genes in Δ*hapR* were lower (ranging from 1-fold to 13-fold). Notably, the increase and decrease patterns for different genes were distinct. For example, at HCD, despite being statistically significant under our statistical tests, the repressive effect of mRNA expression for *vfbH* and *vfbA* was relatively weaker than that for *vfbC* and *vfbB*. When 200 μM 2,2′-bipyridine was added, the expression of *vfbH*, *vfbA*, *vfbC* and *vfbB* were induced (Figures 3C,D) and similar trends were observed in the mRNA expression (1- to 2-fold increase in Δ*aphA* compared to WT and 1- to 14-fold decrease in Δ*hapR* compared to WT).

**Figure 4:**
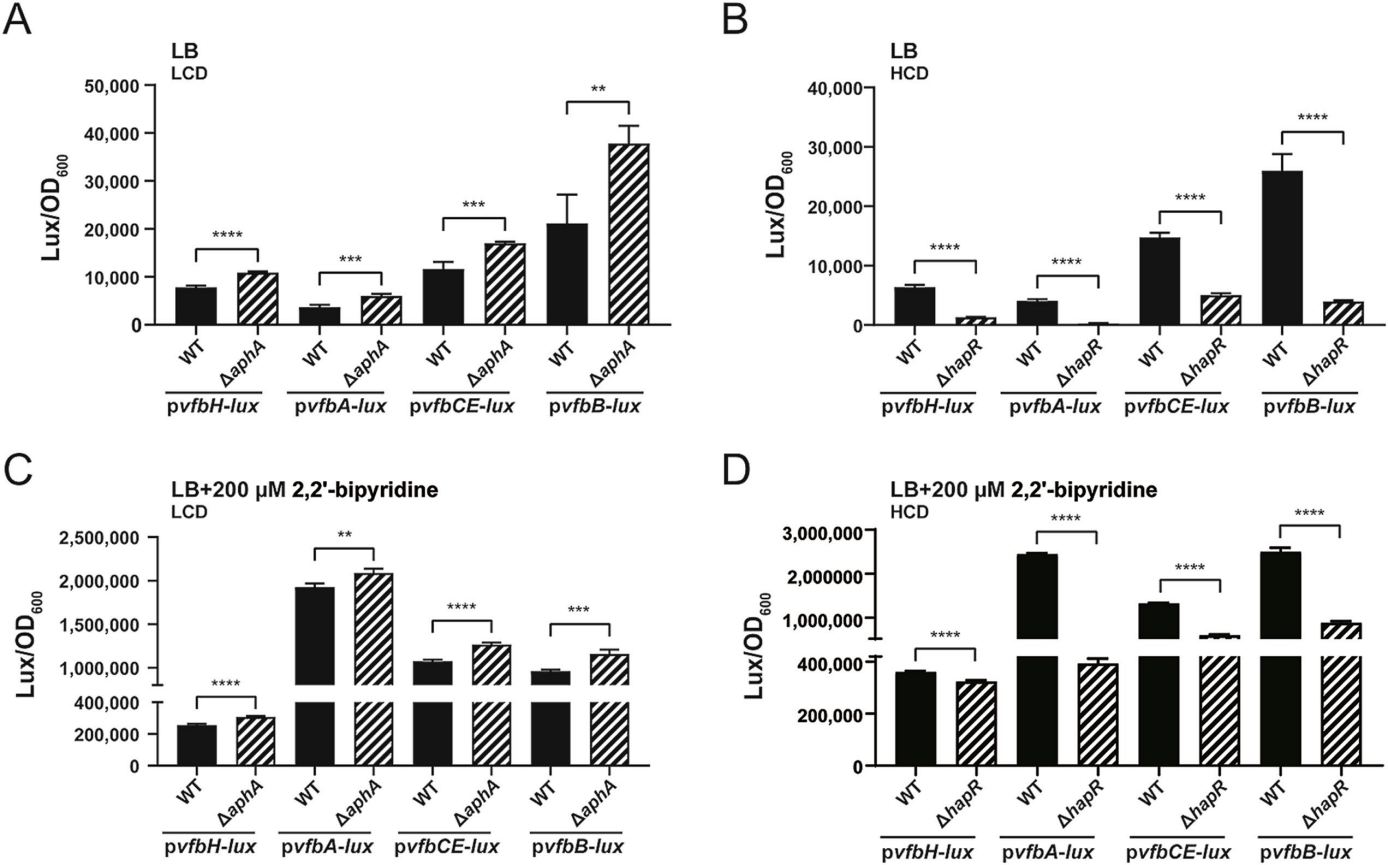
QS regulators AphA and HapR involve in the promoter activities of *vfbH*, *vfbA*, *vfbCE* and *vfbB*. **(A,B)** Promoter activities of *vfbH*, *vfbA*, *vfbCE* and *vfbB* in WT, Δ*aphA* and Δ*hapR* under control LB conditions. **(C,D)** Promoter activities of *vfbH*, *vfbA*, *vfbCE* and *vfbB* in WT, Δ*aphA* and Δ*hapR* when 200 μM 2,2′-bipyridine was added. Luminescence activity assay was performed to examine promoter activities. For Δ*aphA*, the promoter activities at LCD (OD_600_ = 0.2) were examined; for Δ*hapR*, the promoter activities at HCD (OD_600_ = 0.8) were examined; for WT, the promoter activities at both LCD and HCD were examined. Results represented the mean ± SD of three independent biological replicates (*n* = 3). Statistical significance was determined using an unpaired two-tailed Student’s *t*-test. Significant differences were represented as follows: ^**^p < 0.01, ^***^p < 0.001, and ^****^p < 0.0001.

We further investigated the effect of the QS regulators AphA and HapR on the promoter activities of *vfbH*, *vfbA*, *vfbCE* and *vfbB*. The promoterless pBBR-*lux* vector with *luxCDABE* reporter gene was used to construct fusion reporter plasmids p*vfbH*-*lux*, p*vfbA*-*lux*, p*vfbCE*-*lux* and p*vfbB*-*lux*, each of which contains the corresponding promoter region. These recombinant plasmids were then individually transformed into WT, Δ*aphA* or Δ*hapR*, respectively. As shown in Figures 4A,B, under control LB conditions, compared to WT, the luminescence activities of p*vfbH*-*lux*, p*vfbA*-*lux*, p*vfbCE*-*lux* and p*vfbB*-*lux* were significantly higher in Δ*aphA* at LCD, but significantly lower in Δ*hapR* at HCD. When 200 μM 2,2′-bipyridine was added, as shown in Figures 4C,D, compared to WT, the luminescence activities of these recombinant plasmids in Δ*aphA* and Δ*hapR* showed similar trends as those in Figures 4A,B. Together, these results showed at LCD, AphA represses the promoter activities and expression of *vfbH*, *vfbA*, *vfbCE* and *vfbB*, while at HCD, HapR promotes the promoter activities and expression of these genes.

### 3.4 QS regulators AphA and HapR can directly bind to the promoter region of *vfbH*

**Figure 5:**
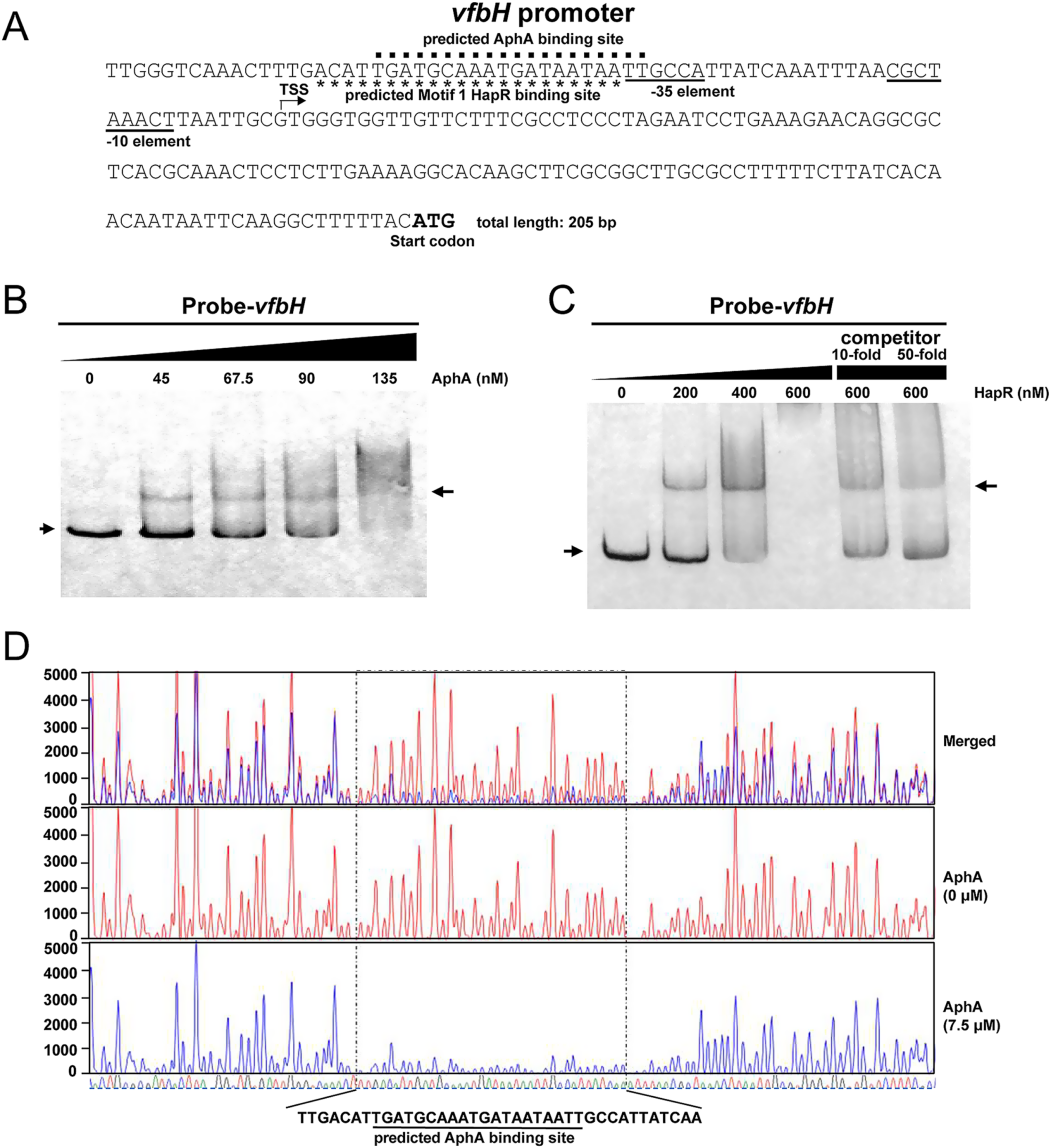
AphA and HapR can directly bind to the promoter region of *vfbH*. **(A)** Genetic map of the region upstream of *vfbH*. The predicted AphA and HapR binding sites were indicated by dots and asterisks, respectively. The putative-35 and -10 promoter elements were underlined. The transcription start site identified by 5′ RACE was indicated by an arrow. The ATG initiation codon of *vfbH* was indicated. **(B,C)** EMSA was performed to examine the binding of AphA (right) or HapR (left) to the promoter region of *vfbH*. The arrow on the left indicated the band of free probes, and the arrow on the right indicated the band of AphA/HapR-bound probes. For HapR, competition analysis was performed by additionally adding 10- or 50-fold unlabeled *vfbH* probes relative to the labeled *vfbH* probes. **(D)** DNase I footprinting assay was performed to examine the AphA binding sequences on the promoter region of *vfbH*. The undigested region protected by AphA was marked by dotted line. The red and blue sequence traces represented the sequences detected at different concentrations of AphA (0 μM and 7.5 μM, respectively). The four-color sequence traces at the bottom represented the region upstream of *vfbH*, with different colors indicating different types of nucleotides. The predicted AphA binding site in panel **(A)** was indicated.

We further explored whether AphA and HapR can directly bind to the promoter regions of *vfbH*, *vfbA*, *vfbCE* and *vfbB* genes. We initially analyzed the potential binding site of AphA or HapR on the sequence of *vfbH* promoter. The amino acid sequences of AphA and HapR in *V. fluvialis* and *V. cholerae* show homologies (for example, the amino acid homologies of AphA and HapR in *V. fluvialis* 85003 and *V. cholerae* O395 are up to 93.3 and 75.36%, respectively). Therefore, the reported consensus binding sequences of AphA (Kovacikova and Skorupski, 2001; Kovacikova et al., 2003; Blokesch et al., 2019) and HapR (including Motif 1 and 2) (Lin et al., 2005; Tsou et al., 2009) in *V. cholerae* were used as references. Based on these consensus binding sequences, one AphA binding site (5′-TGATGCAAATGATAATAATT-3′) and one Motif 1 HapR binding site (5′-ACATTGATGCAAATGATAATAA-3′) were predicted on the promoter region of *vfbH* (Figure 5A). Electrophoretic mobility shift assay (EMSA) and DNase I footprinting assay were performed with 5′ biotin-labeled or fluorescent FAM-labeled probes containing the *vfbH* promoter and purified AphA proteins. As shown in Figure 5B, for AphA, a shifted band was observed in EMSA when 45 nM purified AphA proteins were added. The band for free probes gradually weakened as the concentration of purified AphA proteins increased. DNase I footprinting assay revealed an undigested AphA-protected region, which is a 38 bp sequence of TTGACAT-N_24_-TTATCAA (Figure 5D), which covered the predicted AphA binding site shown in Figure 5A.

For HapR, EMSA with or without unlabeled probes was performed. As shown in Figure 5C, a shifted band was observed in EMSA when 200 nM purified HapR proteins were added and became more apparent when the concentration reached 400 nM. No band was observed when 600 nM purified HapR proteins were added, probably because the bound probes were stuck in the gel well and thus failed to migrate. However, when 10-fold unlabeled probes were additionally added to compete for the labeled probes, the shifted band for bound probes and the lower band for unbound probes appeared. Also, the shifted band for bound probes became less apparent and the lower band for unbound probes became more apparent when the amount of unlabeled competitive probes reached 50-fold. Together, these results suggested that AphA and HapR can directly bind to the promoter region of *vfbH*.

### 3.5 QS regulators AphA and HapR can directly bind to the promoter region of *vfbA/vfbCE*

**Figure 6:**
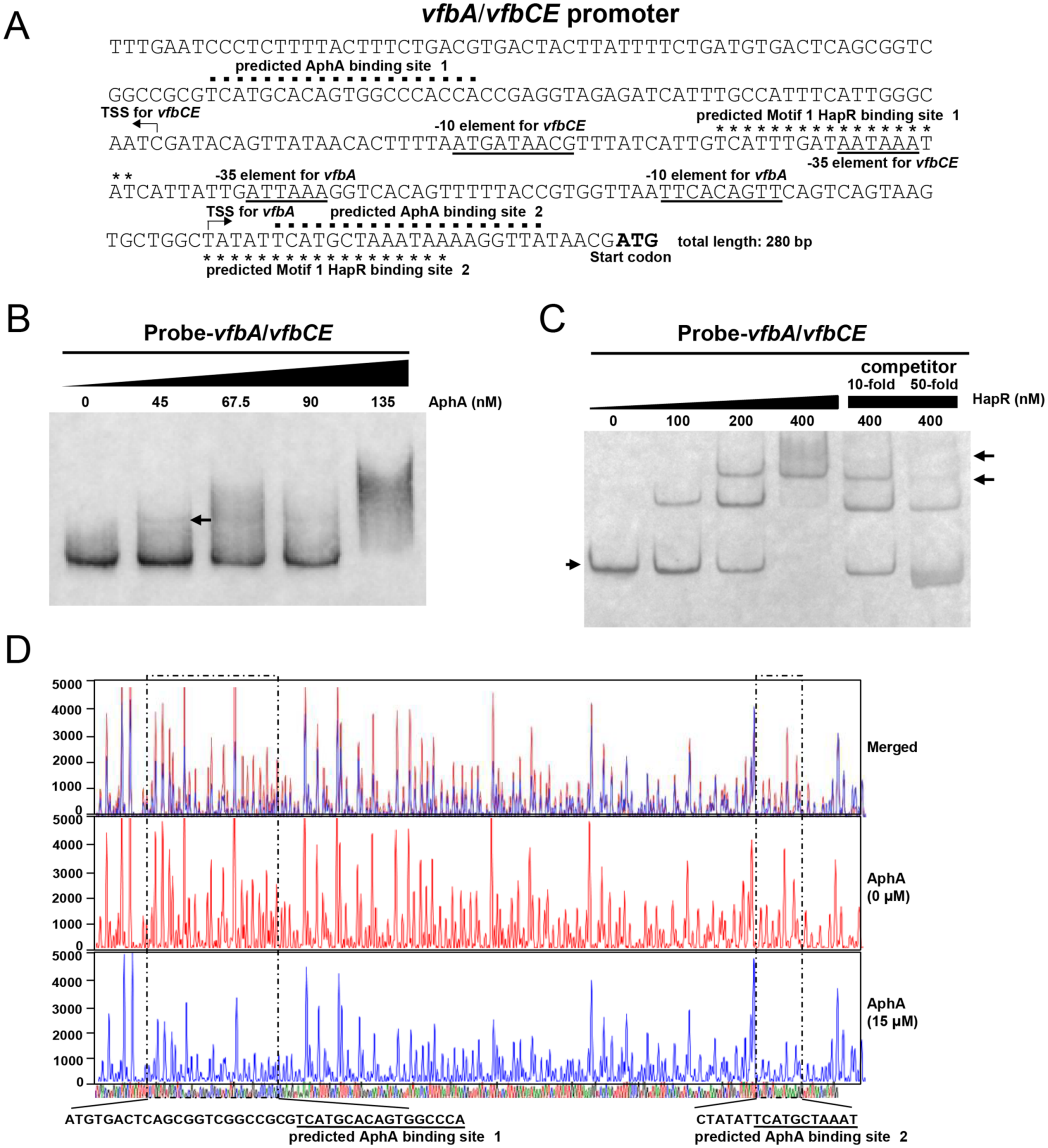
AphA and HapR can directly bind to the promoter region of *vfbA/vfbCE*. **(A)** Genetic map of the intergenic region between *vfbA* and *vfbCE*. For clarity, the sequence orientation of the upstream region of *vfbA* was shown. The predicted AphA and HapR binding sites were indicated by dots and asterisks, respectively. The putative-35 and -10 promoter elements were underlined. The transcription start site identified by 5′ RACE was indicated by arrows. The ATG initiation codon of *vfbA* was indicated. **(B,C)** EMSA was performed to examine the binding of AphA (right) or HapR (left) to the promoter region of *vfbA/vfbCE*. The arrow on the left indicated the band of unbound probes, and the arrow on the right indicated the band of AphA/HapR-bound probes. For HapR, competition analysis was performed by additionally adding 10- or 50-fold unlabeled *vfbA/vfbCE* probes relative to the labeled *vfbA/vfbCE* probes. **(D)** DNase I footprinting assay was performed to examine the AphA binding sequences on the intergenic region between *vfbA* and *vfbCE*. The undigested regions protected by AphA were marked by dotted line and the nucleotide positions relative to the start codon of *vfbA* were labeled. The red and blue sequence traces represented the sequences detected at different concentrations of AphA (0 μM and 15 μM, respectively). The four-color sequence traces at the bottom represented the intergenic region of *vfbA* and *vfbCE* (for clarity, the sequence orientation of the upstream region of *vfbA* was shown), with different colors indicating different types of nucleotides. The predicted AphA binding sites in panel **(A)** were indicated.

As shown in Figures 1A,B, the genes *vfbA* and *vfbCE* belong to different transcription units and their promoters are the same in sequence but opposite in orientation. We further explored whether AphA and HapR can directly bind to the promoter region of *vfbA* or *vfbCE*. As shown in Figure 6A, two AphA binding sites (AphA binding site 1: 5′-TCATGCACAGTGGCCCACCA-3′; AphA binding site 2: 5′-TCATGCTAAATAAAAGGTTA-3′) and two Motif 1 HapR binding sites (Motif 1 HapR binding site 1: 5′-TCATTTGATAATAAATAT-3′; Motif 1 HapR binding site 2: 5′-TATATTCATGCTAAATAA-3′) were predicted on the intergenic region between *vfbA* and *vfbCE* (for clarity, the “5′” and “3′” above indicated the sequence orientation of the upstream region of *vfbA*).

Similarly, EMSA and DNase I footprinting assays were performed to examine the binding between AphA and the promoter region of *vfbA*/*vfbCE*. As shown in Figure 6B, when purified AphA proteins were added, an unapparent shifted band was observed. On the contrary, the band intensity for unbound free probes weakened as the amount of purified AphA proteins increased. DNase I footprinting assay revealed two undigested AphA-protected regions (Figure 6D), which support the binding of AphA to the promoter of *vfbA*. The sequences of the two undigested AphA-protected regions are as follow: ATGTGAC-N_26_-TGGCCCA (40 bp) and CTATATTCATGCTAAAT (17 bp), both of which showed overlaps with the predicted AphA binding sites in Figure 6A.

For HapR, EMSA with or without unlabeled probes was performed as above. As shown in Figure 6C, one shifted band was observed when 100 nM purified HapR proteins were added, and a second shifted band appeared when the amount of purified HapR proteins reached 200 nM. When the amount of purified HapR proteins reached 400 nM, only the second shifted band remained. However, when 10-fold unlabeled probes were additionally added into the 400 nM purified HapR proteins, two shifted bands and the band for unbound probes were observed. When the amount of unlabeled competitive probes reached 50-fold, the two shifted bands for bound probes became less apparent while the band for unbound probes became more apparent. Together, these results suggested that AphA and HapR can directly bind to the promoter region of *vfbA/vfbCE*.

### 3.6 QS regulator AphA can directly bind to the promoter region of *vfbB*

**Figure 7:**
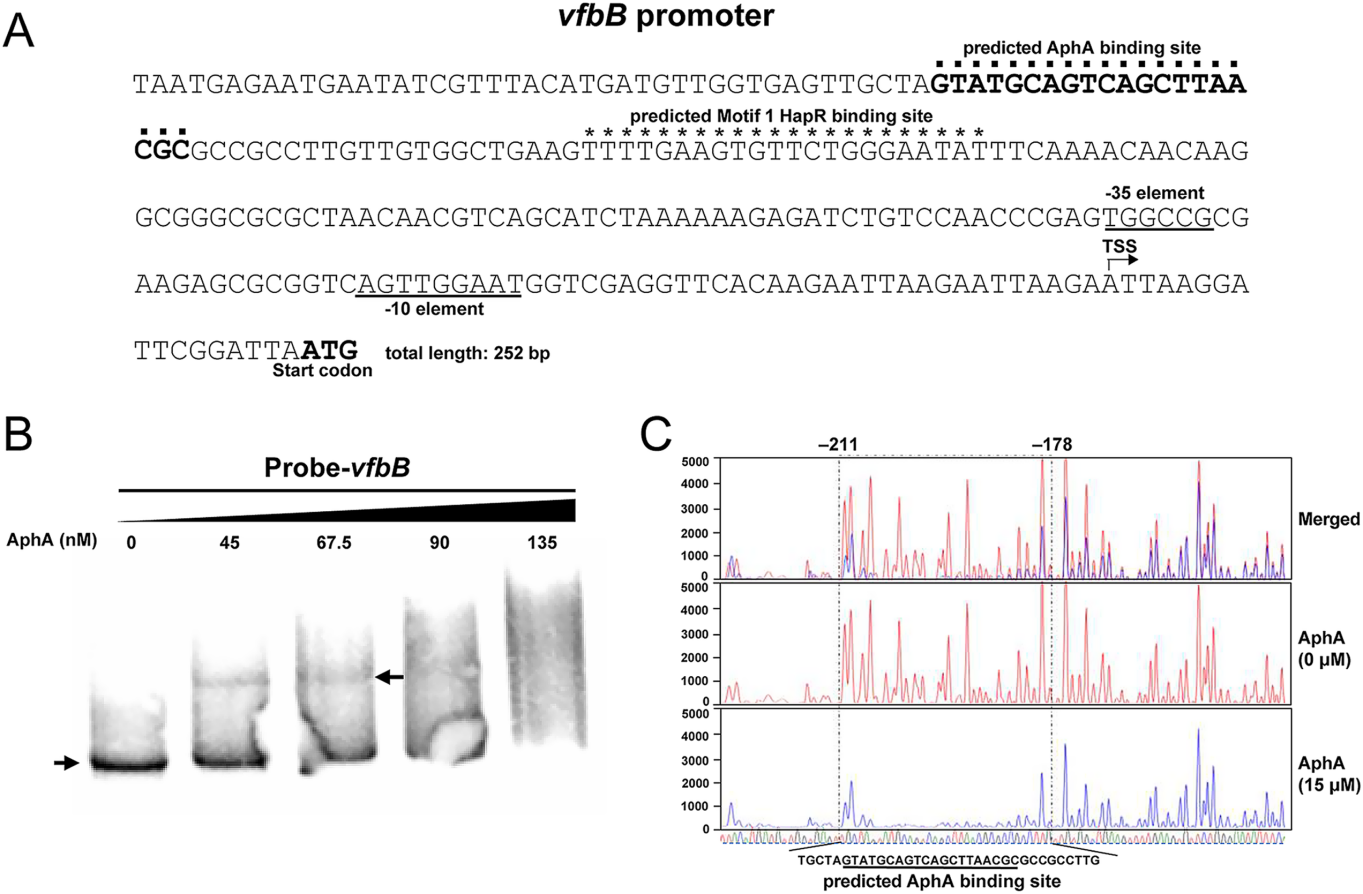
AphA can directly bind to the promoter region of *vfbB*. **(A)** Genetic map of the region upstream of *vfbB*. The predicted AphA and HapR binding sites were indicted by dots and asterisks, respectively. The putative-35 and -10 promoter elements were underlined. The transcription start site identified by 5′ RACE was indicated by an arrow. The ATG initiation codon of *vfbB* was indicated. **(B)** EMSA was performed to examine the binding of AphA to the promoter region of *vfbB*. The arrow on the left indicated the band of unbound probes, and the arrow on the right indicated the band of AphA-bound probes. **(C)** DNase I footprinting assay was performed to examine the AphA binding sequences on the promoter region of *vfbB*. The undigested region protected by AphA was marked by dotted line and the nucleotide positions relative to the start codon of *vfbB* were labeled. The red and blue sequence traces represented the sequence detected at different concentrations of AphA (0 μM and 15 μM, respectively). The four-color sequence traces at the bottom represented the promoter sequence of *vfbB*, with different colors indicating different types of nucleotides. The predicted AphA binding site in panel **(A)** was indicated.

We also explored whether AphA and HapR can directly bind to the promoter region of *vfbB*. Similarly, we predicted the potential binding site for AphA or HapR on the promoter sequence of *vfbB*. As shown in Figure 7A, one AphA binding site (5′-GTATGCAGTCAGCTTAACGC-3′) and one Motif 1 HapR binding site (5′-TTTTGAAGTGTTCTGGGAATAT-3′) were predicted.

Similarly, EMSA and DNase I footprinting assays were performed to examine the binding between AphA and the promoter region of *vfbB*. As shown in Figure 7B, a shifted band was observed when 45 nM purified AphA proteins were added. DNase I footprinting assay also revealed an undigested AphA-protected region, which is a 34 bp sequence (TGCTAGT-N_20_-CGCCTTG), as shown in Figure 7C. This region includes the predicted AphA binding site shown in Figure 7A. For HapR, no shifted band was observed by EMSA (data not shown), which may due to the low binding affinity and needs further exploration. Together, these results suggested that AphA can directly bind to the promoter region of *vfbB*.

### 3.7 Deletion of *vfbACEB* causes decreased bacterial loads in mice

**Figure 8:**
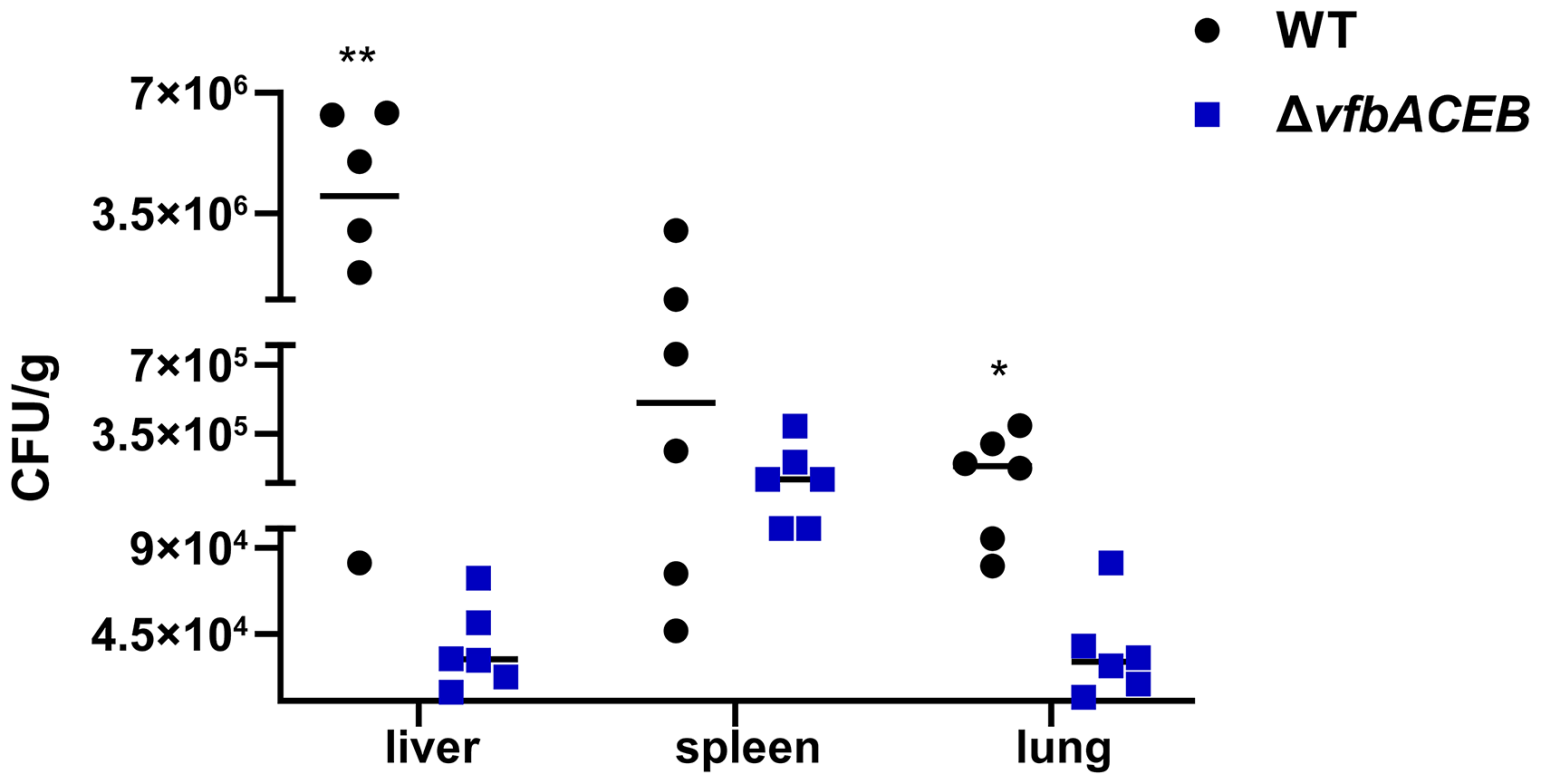
Deletion of *vfbACEB* causes decreased bacterial loads in mice. Female C57BL/6 mice (*n* = 6 for each group) were infected intraperitoneally with WT or Δ*vfbACEB* (2 × 10^9^ CFU for each). At 12 h post-infection, the liver, spleen, and lung samples of infected mice were collected after euthanasia, before calculating the bacterial loads in these samples. Horizontal lines represent the median value of each group (*n* = 6 mice per group). Statistical significance was determined using an unpaired two-tailed Student’s *t*-test. Significant differences were represented as follows: ^*^p < 0.05 and ^**^p < 0.01.

In bacteria, siderophores are considered as critical virulence factors which contribute to the pathogenesis of bacterial infection through promoting iron acquisition and facilitating bacterial survival and proliferation within the host environment (Holden and Bachman, 2015). We assumed that the failure to biosynthesize fluvibactin and the inability to acquire iron would hamper the dissemination and survival of *V. fluvialis* in visceral organs (for example, liver, spleen, and lungs), where host nutritional immunity tightly sequesters free iron (Ganz and Nemeth, 2015; Murdoch and Skaar, 2022). Therefore, we evaluated the effect of fluvibactin biosynthesis on *V. fluvialis* systematic infection. Female C57BL/6 mice (*n* = 6 for each group, *n* = 12 in total) were infected intraperitoneally with WT or Δ*vfbACEB* (2 × 10^9^ CFU for each). At 12 h post-infection, the liver, spleen and lung samples of infected mice were collected to examine bacterial burden. As shown in Figure 8, the bacterial loads in Δ*vfbACEB* were lower than those in WT, especially in the liver samples where the most significant differences were observed (the median CFU/g in WT = 4.0 × 10^6^; the median CFU/g in Δ*vfbACEB* = 3.2 × 10^4^), followed by the lung (the median CFU/g in WT = 1.9 × 10^5^; the median CFU/g in Δ*vfbACEB* = 3.0 × 10^4^) and the spleen (the median CFU/g in WT = 5.1 × 10^5^; the median CFU/g in Δ*vfbACEB* = 1.2 × 10^5^) samples. Together, these results showed that the abolishment of fluvibactin biosynthesis reduces the bacterial load and colonization capacity of *V. fluvialis* in host tissues.

## 4 Discussion

Fluvibactin is the catechol siderophore produced by *V. fluvialis*, an emerging foodborne pathogen of public health concern (Yamamoto et al., 1993; Igbinosa and Okoh, 2010; Ramamurthy et al., 2014). In this study, we characterized the *vfbABCDEFH* genes for fluvibactin biosynthesis and focused on how the QS regulators AphA and HapR involve in the expression of *vfbABCEH* genes. Our results showed that like the genes for vibriobactin biosynthesis in *V. cholerae* (Wyckoff et al., 1997; Butterton et al., 2000; Keating et al., 2000a, 2000b; Wyckoff et al., 2001), the genes *vfbABCDEFH* for fluvibactin biosynthesis are located in a gene cluster, which not only include *vfbABCDEFH* themselves, but also the genes for fluvibactin transport and with unknown functions, forming nine transcription units in total. We then demonstrated that fluvibactin synthesis is induced by iron limitation and cell density. The QS regulator AphA at LCD represses the expression of *vfbABCEH* genes while the QS regulator HapR at HCD activates their expression. Further, we demonstrated that AphA can directly bind to the promoter regions of *vfbH*, *vfbA/vfbCE* and *vfbB*, while HapR can directly bind to the promoter regions of *vfbH* and *vfbA/vfbCE*. Moreover, the deletion of *vfbABCE*, the product of which participate in the formation of the intermediate DHBA for the subsequent fluvibactin biosynthesis, caused decreased bacterial loads of *V. fluvialis* in infected mice.

The structure and organization of genes for catechol siderophore biosynthesis vary considerably in different *Vibrio* species (Payne et al., 2016). In this study, we found that the homologs of *vibACEB* genes for vibriobactin biosynthesis in *V. cholerae* showed similar distributions in the pathogenic *Vibrio* species, except that one extra gene was found between the *vibB* and *vibE* homologs in *V. vulnificus* and *V. splendidus*, and that one extra gene was found between the *vibA* and *vibC* homologs in *V. harveyi* and *V. campbellii* (Figure 1A). Notably, in *V. harveyi* and *V. campbellii*, the transcriptional orientations of the homologs of *vibA*, *vibC* and *vibE* were found to be opposite to other *Vibrios*. Compared to *vibACEB*, the homologs of *vibD*, *vibH* and *vibF* genes showed various distributions among different *Vibrio* species. For example, the homologs of *vibF*, the product of which acts as a scaffold on which vibriobactin is synthesized (Keating et al., 2000a), were found to show the greatest differences. In *V. cholerae*, *vibF* is a single gene in a separate gene cluster distant from the cluster of *vibABCDEH* (Butterton et al., 2000), whereas in *V. fluvialis*, *V. furnissii*, *V. vulnificus*, *V. splendidus*, *V. harveyi* and *V. campbellii*, the *vibF* homologs are one or two copies in the same cluster as the homologs of *vibABCDE*. Since the length of the gene *vibF* in *V. cholerae* is approximately 7,000 bp (Butterton et al., 2000), its homologs in other *Vibrio* species may consist of several shorter copies linked together or not. Specifically, in *V. fluvialis* and *V. furnissii*, the two *vibF* homologs are separated by seven genes including the three genes for siderophore transport; in *V. splendidus*, the two *vibF* homologs are separated by the three genes for siderophore transport; in *V. vulnificus*, the two *vibF* homologs are linked together, with a 69 bp interval.

Although we demonstrated that under iron-limited conditions, AphA at LCD can repress while HapR at HCD can facilitate fluvibactin synthesis, the repressive effect of AphA and the stimulative effect of HapR are not absolute. Moreover, apart from iron-limited conditions, AphA and HapR can regulate fluvibactin synthesis under control LB conditions. We assumed that other factors, such as the ferric uptake regulator and iron-dependent transcriptional regulator Fur, may work together with these QS regulators to regulate fluvibactin synthesis. Actually, in *V. vulnificus*, the primary QS regulator SmcR binds to the transcription start site of *vvsAB*, which is homologous to the *vibF* in *V. cholerae* and encodes a NRPS required for vulnibactin synthesis. Moreover, the binding site of SmcR on *vvsAB* overlaps with its Fur-binding site (Wen et al., 2012). SmcR and Fur work jointly to regulate vulnibactin biosynthesis. Under high-iron conditions, Fur repressed *vvsAB* transcription, while under low-iron conditions, SmcR regulated the expression of the vulnibactin biosynthesis genes. Specifically, Fur was found to bind at two sites upstream of the transcription start site of *smcR*, repressing the production of SmcR under high-iron environments. However, the affinity of Fur at these sites was so low that *smcR* was induced by QS at HCD, even under iron-rich conditions. Under iron-limited conditions, Fur did not bind and *smcR* expression was regulated solely by QS (Wen et al., 2012). The cross talk and dual control between Fur (iron) and QS regulation may allow the fine-tuning of iron acquisition. Actually, Fur binding sites were predicted on the promoters of *vfbH*, *vfbA*/*vfbCE* and *vfbB*, showing partial overlaps with the binding sites of AphA or HapR. Further studies are needed to explore how Fur work jointly with AphA and HapR in the regulation of fluvibactin biosynthesis, maintaining intracellular iron homeostasis and optimal bacterial survival. The co-regulation by Fur and QS regulator may avoid the excess of intracellular labile irons, which can induce ferroptosis (Huang et al., 2023). Meanwhile, the joint regulation by Fur and QS regulator may influence the availability of environmental iron, which is related to the virulence of bacteria. In *V. parahaemolyticus*, ferric iron can contribute to its intestinal colonization and virulence through the signal pathway of EnvZ/OmpR two-component system (Zhang et al., 2025).

Our study showed that QS promoted fluvibactin synthesis in *V. fluvialis*, similar to the phenomenon observed in *Pseudomonas aeruginosa* (Stintzi et al., 1998). This mode of regulation may ensure sufficient siderophore available for bacterial growth (Darch et al., 2012). Our *in vivo* experiments revealed that fluvibactin biosynthesis enhances the bacterial loads of *V. fluvialis* in infected mice (Figure 8). Previous studies have shown that the hemin-binding outer membrane protein HupO, which also mediates iron acquisition, significantly enhanced the virulence of *V. fluvialis* (Ahn et al., 2005). Considering the important role of iron in *V. fluvialis* pathogenicity, the activation of fluvibactin biosynthesis by QS may benefit the survival and environment adaptation of *V. fluvialis*. However, studies in *V. harveyi* (Lilley and Bassler, 2002) and *V. vulnificus* (Wen et al., 2012) reported that QS inhibits siderophore biosynthesis. The differences may relate to the distinct organization of siderophore biosynthesis genes in these *Vibrio* spp. (Figure 1A). The different regulation of siderophore biosynthesis by QS is likely to affect the adaptability, competitiveness and thus survival advantages of bacteria in the same ecological niche. In *V. cholerae* infected *Drosophila*, the activation of QS has been reported to repress the succinate uptake by *V. cholerae*, resulting in increased host access to intestinal succinate, which undermines the lipid lost caused by infection and promote the survival of infected flies (Kamareddine et al., 2018). Under anaerobic conditions, the ArcAB system of *V. cholerae* promotes biofilm formation by repressing the transcription of *hapR*, which encodes the HCD QS regulator (Caigoy et al., 2025).

Admittedly, there are several limitations in our current study. First, while the *vfbACEB* mutant exhibited reduced bacterial loads in the infected mouse model, the lack of an *in vivo* complemented strain makes it difficult to definitively attribute this reduction solely to a specific virulence defect. The impaired colonization on host tissues may partially reflect a general fitness cost associated with iron starvation in the host. Future studies incorporating complemented strains and additional infection readouts (e.g., cytotoxicity or immune evasion assays) are needed to uncouple general fitness from specific virulence mechanisms. Also, instead of focusing on systemic infection, how fluvibactin biosynthesis influences the intestinal colonization of *V. fluvialis* needs further investigation. Second, the *in vitro* EMSA and footprinting assays demonstrated that AphA and HapR can directly bind to the fluvibactin biosynthesis genes, but the precise *in vivo* mechanisms linking this binding to transcriptional outcomes, such as their interactions with RNA polymerase or other transcriptional co-factors, remain to be fully elucidated. Although the loss of AphA or HapR has been shown to alter the expression of fluvibactin biosynthesis genes, whether the differences are meaningful in a genuine infection context also need further exploration, especially considering that the changes of some genes are relatively small. Finally, the biochemical functions of the *vfb* cluster are inferred from sequence similarity and warrant future enzymatic validation.

In conclusion, our study revealed the gene cluster for fluvibactin biosynthesis and explored the regulatory mechanisms of QS regulators AphA and HapR on fluvibactin biosynthesis genes, which enrich the understandings of siderophore biosynthesis and regulation in pathogenic *Vibrios*, and offer insights into the pathogenicity and environmental adaptation of *V. fluvialis*.

## Data Availability

The datasets presented in this study can be found in Science Data Bank at: https://doi.org/10.57760/sciencedb.28121 (Xiao et al., 2025).

## Footnotes

1
https://github.com/gamcil/clinker
