# Supplementary material for: Quorum sensing regulators AphA and HapR differentially regulate fluvibactin biosynthesis in Vibrio fluvialis
Source: Front Microbiol. 2026 May 4;17:1829491. doi: 10.3389/fmicb.2026.1829491 (PMC13180887; doi:10.3389/fmicb.2026.1829491)
Supplement: Supplementary file 1 [file Data_Sheet_1.pdf]

Supplementary Material

1 Supplementary Figures and Tables

1.1 Supplementary Figure

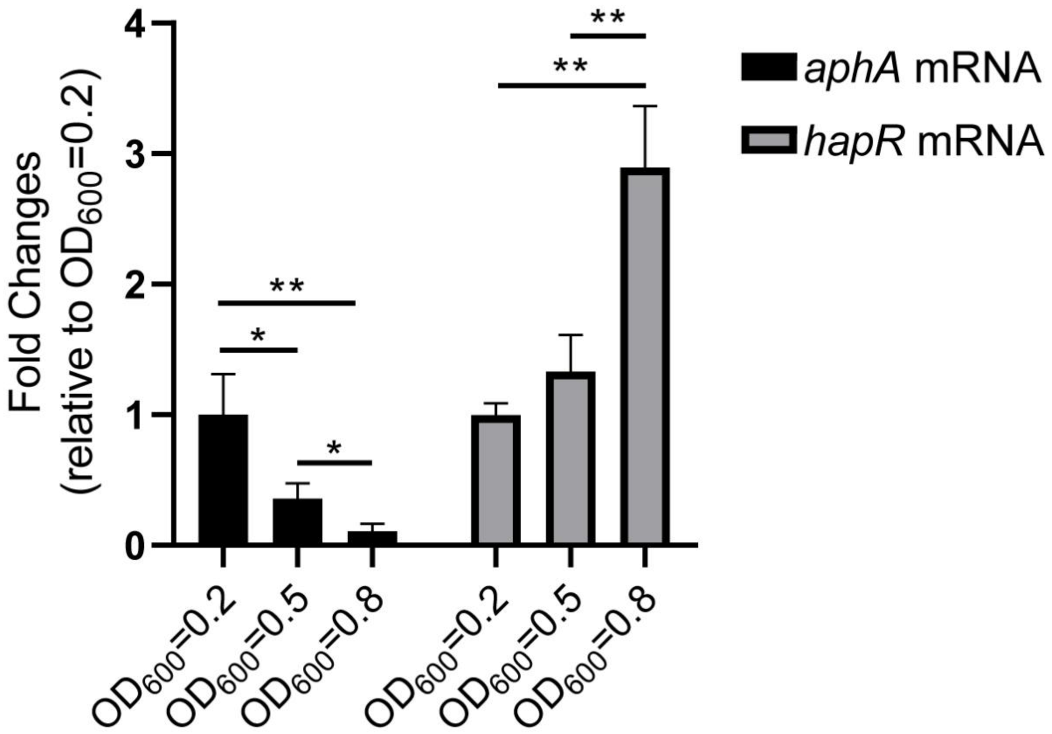

Supplementary Figure S1. AphA is highly expressed at LCD and HapR is highly expressed at HCD. Relative mRNA levels of *aphA* and *hapR* in WT under different cell densities (OD_{600} = 0.2, 0.5, 0.8) examined by qPCR. The mRNA levels at OD_{600} = 0.2 were used as internal control and the fold changes at different cell densities were represented. Results represented the mean ± SD of three independent experiments. Significant differences were represented as follows: *, $P< 0.05$; **, $P < 0.01$.

1.2 Supplementary Tables

Supplementary Table S1. Bacteria strains and plasmids used in this study.

Bacterial strains/plasmids	Characteristics	Sources
**Bacterial strains**		
Escherichia coli		
SM10*λpir*	*thr thi tonA leu supE lacY recA*::RP4-2Tc::Mu (*λpir* R6K)，Km^R	Mekalanos Laboratory (Harvard Medical School)
Top10	F- *mcrA* Δ *(mrr-hsd RMS-mcrBC) φ*80*lacZ*Δ*M*15Δ*lacX*74 *recA*1 *deoRara*Δ139Δ*(ara-leu)*7697 *galU galK rpsL* (*StrR*) *endA*1 *nupG*	Laboratory stock
DH5α*λpir*	*recA1 endA1 hsdR17 thi-1 gyrA96 relA*1*λpir*	Laboratory stock
BL21(DE3)	F- *ompT hsdS*$_B$ (r_B- m_B-) *gal dcm* (DE3) pRARE2, Cm^R	Laboratory stock
Vibrio fluvialis		Laboratory stock
85003	wild type (WT), Sm^R	Laboratory stock
Δ*aphA*	*aphA* in-frame deletion strain of 85003, Sm^R	(Cheng et al., 2024)
Δ*hapR*	*hapR* in-frame deletion strain of 85003, Sm^R	(Wang et al., 2013)
Δ*vfbACEB*	*vfbACEB* in-frame deletion strain of 85003, Sm^R	This study
**Plasmids**		
pWM91	suicide vector containing R6 K ori, *sac*B, *lacZ*α; Amp^R	Laboratory stock
pBBR*lux*	bioluminescence based reporter plasmid containing a promoterless *luxCDABE* operon, CmR	Laboratory stock
pET28a	expression vector containing f1 ori, *lacI*, Km^R	Laboratory stock
pET30a	expression vector containing f1 ori, *lacI*, Km^R	Laboratory stock
pWMΔ*vfbACEB*	*vfbACEB* in-frame deletion fragment of 85003 in pWM91	This study
p*vfbH-lux*	pBBR*lux* with the promoter region of *vfbH* (202 bp) of 85003	This study
p*vfbA-lux*	pBBR*lux* with the promoter region of *vfbA* (277 bp) of 85003	This study
p*vfbCE-lux*	pBBR*lux* with the promoter region of *vfbCE* (277 bp) of 85003	This study
p*vfbB-lux*	pBBR*lux* with the promoter region of *vfbB* (249 bp) of 85003	This study
pET*aphA*	pET28a with the *aphA* ORF (540 bp) of 85003	(Cheng et al., 2024)
pET*hapR*	pET30a with the *hapR* ORF (615 bp) of 85003	(Liu et al., 2021)

Supplementary Table S2. Primers used in this study.

Primer name	Sequences(5′ ~ 3′)
Δ*vfbACEB*-F1-F-XhoI	GCGAATTGGGTACCGGGCCCCCCCTCGAGGATCTCGATGGCGCACATAAC
Δ*vfbACEB*-F1-R	GAATTAAGGATTCGGATTAATGTGAGGCCTTTCACACACATAAG
Δ*vfbACEB*-F2-F	TTATGTGTGTGAAAGGCCTCACATTAATCCGAATCCTTAATTCTTAATTC
Δ*vfbACEB*-F2-R-SacI	TCACTAAAGGGAACAAAAGCTGGAGCTCGAGTGAAAACTTCACCACCTTC
A I-F	CCACACTGCTCATGGTGC
A I-R	GATCACCGACAACGATGTG
A II- F	TTGGCACGGTTGATGATGC
A II-R	GTCGAGAGGGGCTCATCC
A III-F	GAATGGGTGGTGACCATCC
A III-R	CACGCCAATTTGCAGCAG
A IV-F	AATGCCAGCTTGTTTAGCCG
A IV-R	CTGCCTTTTTTATTGTCCGC
AL536_11480-qPCR-F	CGCGCACTGATGTAGAACGG
AL536_11480-qPCR-R	CACGGTCGGTGTGATGGTCG
vfuP-qPCR-F	GCAGCATGCCGTGTGAAC
vfuP-qPCR-R	GCGGCCAAAACTGGCAAC
AL536_11460-qPCR-F	GCTGCCGGGGTTGTCTCTC
AL536_11460-qPCR-R	ATATTCGGGGTGGGGCAGC
vfbH-qPCR-F	GGAGATCAAAGATCTGCGCGG
vfbH-qPCR-R	CTGCTCAAACAGCGTCATGCC
vfbF1-qPCR-F	GCCAACTCACTCAACAGCGC
vfbF1-qPCR-R	CACCTCGGGCTGGGACATC
AL536_11445-qPCR-F	CGAAGCAAGGGCTTGAATACGC
AL536_11445-qPCR-R	CCTAAATCGGTCACGTCACGC
vfbA-qPCR-F	GCGGGATCCCCAGTCGATAG
vfbA-qPCR-R	CCCGAATATGGGGGCGTATGG
vfbC-qPCR-F	GCTTGCGATCAATGCCAAAGG
vfbC-qPCR-R	CTACCTCTTCCACCACCAAGCC
vfbB-qPCR-F	GACAGCGCTTCAGCCACATC
vfbB-qPCR-R	GCGTATACGGCCACATCGG
pro-*vfbH*-F-SacI	CGGAGCTCCACTGGATTGTGATTGGCAC
pro-*vfbH*-R-BamHI	CGGGATCCGTAAGGGGCATCCAATGAAG
pro-*vfbA*-F-SacI	CGGAGCTCGCTCTCCATTTTTGTATAGCC
pro-*vfbA*-R-BamHI	CGGGATCCGTTTTATCCGTCTGGGTCAC
pro-*vfbCE*-F-SacI	CGGAGCTCGTTTTATCCGTCTGGGTCAC

Primer name	Sequences(5′ ~ 3′)
pro-*vfbCE*-R-BamHI	CGGGATCCGCTCTCCATTTTTGTATAGCC
pro-*vfbB*-F-SacI	CGGAGCTCGTTGCTGATTGTGACCTTGC
pro-*vfbB*-R-BamHI	CGGGATCCCAATCTGCCACTGAGTTTTG
*vfH*_5′ RACE-GSP	**GATTACGCCAAGCTT**GCATCCGCCAGTGGC TGCGAACTGC
*vfH*_5′ RACE-NGSP	**GATTACGCCAAGCTT**ATCTCCACCTGCGGTA TGCGCG
*vfbA*_5′ RACE-GSP	**GATTACGCCAAGCTT**GTTGTCGAGCGTCGC GCCTCCGTCA
*vfbA*_5′ RACE-NGSP	**GATTACGCCAAGCTT**GTGATGTGGTTGGCC GCATCCGA
*vfCE*_5′ RACE-GSP	**GATTACGCCAAGCTT**CGCTTGGTTGGCCGC CGTGTTGGC
*vfCE*_5′ RACE-NGSP	**GATTACGCCAAGCTT**GCTGCTGCGGAATCA CAAATCGGGT
*vfbB*_5′ RACE-GSP	**GATTACGCCAAGCTT**TGCCATGGCAGGTTT CGCCTCCGTTCG
*vfB*_5'RACE-NGSP	**GATTACGCCAAGCTT**CACCACTCCTGCAAC GTCACTTTCTC
EMSA-F (Biotion-labeled/unlabeled)	GACTCACTATAGGGCGAATTGG
EMSA-R (Biotion-labeled/unlabeled)	TTGCCATCCATTTTGCGGC
vfbH-F (FAM-labeled)	GTGATTGGGTCAAACTTTGAC
vfbH-R	ACTGACATGTAAAAAGCCTTG
vfbA-F (FAM-labeled)	GACTCACTATAGGGCGAATTGG
vfbA-R	TTGCCATCCATTTTGCGGC
vfbB-F (FAM-labeled)	ATAATAATGAGAATGAATATCGTTTACATG
vfbB-R	GATTGCCATTAATCCGAATCC

[a]: The underlined nucleotides indicate corresponding restriction endonuclease sites.

[b]: The bold nucleotides indicate the sequence on pRACE vector for in-fusion homology.

2 References

Cheng, Q., Han, Y., Huang, Y.M., Ji, S.S., Li, J., Diao, B.W., et al. (2024). [Regulation mechanism of the quorum sensing regulator AphA on the type Ⅵ secretion system VflT6SS2 in Vibrio fluvialis]. *Zhonghua Liu Xing Bing Xue Za Zhi* 45(4)**,** 566-573. doi: 10.3760/cma.j.cn112338-20231215-00354.

Liu, X., Pan, J., Gao, H., Han, Y., Zhang, A., Huang, Y., et al. (2021). CqsA/LuxS-HapR Quorum sensing circuit modulates type VI secretion system VflT6SS2 in *Vibrio fluvialis*. *Emerging Microbes & Infections* 10(1)**,** 589-601. doi: 10.1080/22221751.2021.1902244.

Wang, Y., Wang, H., Liang, W., Hay, A.J., Zhong, Z., Kan, B., et al. (2013). Quorum Sensing Regulatory Cascades Control *Vibrio fluvialis* Pathogenesis. *Journal of Bacteriology* 195(16)**,** 3583-3589.
